# Name recognition in autism: EEG evidence of altered patterns of brain activity and connectivity

**DOI:** 10.1186/s13229-016-0102-z

**Published:** 2016-09-06

**Authors:** Anna Nowicka, Hanna B. Cygan, Paweł Tacikowski, Paweł Ostaszewski, Rafał Kuś

**Affiliations:** 1 Laboratory of Psychophysiology, Department of Neurophysiology, Nencki Institute of Experimental Biology, Polish Academy of Sciences, 3 Pasteur Street, 02-093 Warsaw, Poland; 2 Central Institute for Labour Protection - National Research Institute, Czerniakowska 16, 00-701 Warsaw, Poland; 3 Brain, Body, and Self Laboratory, Department of Neuroscience, Karolinska Institute, Retzius väg 8, SE-17177 Stockholm, Sweden; 4 Department of Psychology, University of Social Sciences and Humanities, 19/31 Chodakowska Street, 03-815 Warsaw, Poland; 5 Faculty of Physics, University of Warsaw, 5 Pasteur Street, 02-093 Warsaw, Poland

**Keywords:** Autism spectrum disorder, Coherence, ERP, Directed transfer function, Event-related desynchronization and synchronization

## Abstract

**Background:**

Impaired orienting to social stimuli is one of the core early symptoms of autism spectrum disorder (ASD). However, in contrast to faces, name processing has rarely been studied in individuals with ASD. Here, we investigated brain activity and functional connectivity associated with recognition of names in the high-functioning ASD group and in the control group.

**Methods:**

EEG was recorded in 15 young males with ASD and 15 matched one-to-one control individuals. EEG data were analyzed with the event-related potential (ERP), event-related desynchronization and event-related synchronization (ERD/S), as well as coherence and direct transfer function (DTF) methods. Four categories of names were presented visually: one’s own, close-other’s, famous, and unknown.

**Results:**

Differences between the ASD and control groups were found for ERP, coherence, and DTF. In individuals with ASD, P300 (a positive ERP component) to own-name and to a close-other’s name were similar whereas in control participants, P300 to own-name was enhanced when compared to all other names. Analysis of coherence and DTF revealed disruption of fronto-posterior task-related connectivity in individuals with ASD within the beta range frequencies. Moreover, DTF indicated the directionality of those impaired connections—they were going from parieto-occipital to frontal regions. DTF also showed inter-group differences in short-range connectivity: weaker connections within the frontal region and stronger connections within the occipital region in the ASD group in comparison to the control group.

**Conclusions:**

Our findings suggest a lack of the self-preference effect and impaired functioning of the attentional network during recognition of visually presented names in individuals with ASD.

**Electronic supplementary material:**

The online version of this article (doi:10.1186/s13229-016-0102-z) contains supplementary material, which is available to authorized users.

## Background

Autism spectrum disorder (ASD) is a heterogeneous neurodevelopmental disorder characterized by impaired social interactions and communication, restricted interests, and repetitive behaviors. According to the various data sources the prevalence of ASD is estimated at 1 in 160 children (WHO: www.who.int) up to 1 in 68 children (CDC: www.cdc.gov). This high prevalence makes ASD an important problem, not only for the affected individuals and their families, but also for the society in general. Despite extensive research in the field, the etiology of ASD remains unknown and there is still no unified theory that would explain all autistic symptoms or suggest clear guidelines for treatment [1–4].

Many existing studies have tried to understand the autistic mind by analyzing behavioral and neural responses during the processing of various information that are crucial in social interactions, e.g. gaze direction and other people’s intentions [5, 6]. In particular, the topic of impaired face processing in ASD has been extensively studied (for reviews see [7–10]). Given the fact that both faces and names are typical cues prompting interpersonal interactions, investigation of proper names processing may provide new insights into the nature of the autistic mind. However, this topic has not gained enough attention so far and the neural mechanisms of name processing in ASD remain unknown.

In non-ASD populations, studies on name processing have consistently shown the preferential status of the self-name (e.g., [11–15]). This stimulus is easily selected among other information, i.e., the so-called cocktail party effect [16, 17] and enters awareness more easily than other types of emotional and social stimuli [18–20]. Even 5-month-old infants recognize their own name and use it as a social cue to guide their attention to events and objects in the external world [21–23]. Preferential processing of one’s own name has a clear adaptive value. In every-day life, our name is often called to attract our attention to the speaker; it may signal some potentially important information (e.g., a warning) but even more often it is used to initiate social interaction. Because this happens countless times throughout the lifespan, people probably start to respond to this stimulus in a highly preferential and automatic manner. It has been also proposed that one’s own name is the core element of social identity and knowledge about oneself; our own name and surname define us as individuals among other people [24, 25].

As mentioned earlier, little is known about name processing in ASD but the limited existing evidence suggests that it is atypical. Retrospective analyses of home videos reveal that autistic children often show reduced reaction or a lack of reaction to their own name; crucially, this symptom is present long before the time of the actual clinical diagnosis [4, 5, 26, 27]. To the best of our knowledge, only two studies have investigated the neural correlates of name processing in ASD. The first study investigated passive listening to one’s own name [28]. Such procedure resulted in the right medial and middle frontal gyri activations. However, those results are rather difficult to interpret because the experiment was done on a single sedated individual with ASD and it lacked crucial control conditions (other names); thus, this study does not inform on the presence/absence of the preferential processing of the self-name in ASD. The second study [29] was conducted by our group and investigated event-related potentials (ERPs) in high-functioning adolescents and adults with ASD during detection of visually presented names: one’s own, close-other’s, famous, and unknown. It was found that P300 (the late ERP component with an approximate latency of 300 ms after stimulus onset) in the ASD group did not differ between the processing of self-name and close-other’s name, whereas in the control group this difference was significant [29]. Given that P300 response is often seen as an electrophysiological index of sustained attention and allocation of cognitive resources [30], this P300 effect suggests that even high-functioning adult individuals with ASD still show reduced attentional/orienting response to their own name.

Reduced attention to self-name in individuals with ASD fits well with the social motivation theory of autism, which posits that these individuals generally fail to “affectively tag” socially relevant stimuli as intrinsically rewarding, thus, responding to such stimuli does not become attentionally preferential [4, 31]. What remains unknown, however, is the type of attention which leads to atypical name processing; in our previous study we used a simple detection task which mainly involves bottom-up attention [29]. Although models of selective attention propose the existence of two distinct processes, reflecting distinct operations: a bottom-up, stimulus-driven (i.e., exogenous) and a top-down, goal-driven (i.e., endogenous) attention [32], it is generally accepted that they interact with one another to guide selective attention [33]. Thus, one may wonder whether impaired—on the neural level—differentiation of one’s own name from a close-other’s name in individuals with ASD would still be present in the case of enhanced involvement of internally-guided, top-down attention. In order to resolve this issue we decided to investigate the name recognition in the ASD and control groups. Crucially, in comparison to the detection task, the explicit name recognition involves higher order processing and requires the involvement of internally guided, top-down attention [32]. Similarly to our previous study [29], names were presented in the visual modality.

In addition, it is also an unknown whether social attention deficits in ASD are indeed specific to self-name processing only or occur more generally for other types of names; findings from a single study [29] are not enough to answer this question. Finally, it is an unresolved issue whether in this clinical group, other—than P300—markers of attention would reveal any deficits related to the name recognition task. Investigation of different electrophysiological markers of attention during a name recognition task could provide important evidence regarding social motivation/social attention deficits in autism. The two candidates for such additional electrophysiological markers of attention were alpha suppression and beta synchronization.

Briefly, modulation of alpha power reflects processes that regulate information flow in the cortex via selective suppression and selection of sensory signals [34–36]. Alpha suppression occurs when attention is directed towards external stimuli [37] and—following sensory stimulation—can be observed in the corresponding sensory areas [38]. Stimuli to-be-ignored are associated with increased alpha synchronization [34, 39]. Moreover, lower alpha suppression is a likely indicator of weakened top-down control [40–42]. Beta oscillations, in turn, are usually associated with alertness and active task engagement [43] and are viewed as a “carrier” of visual attention in humans [44–47]. For instance, only successful visual discrimination is associated with the increase of beta band power recorded from occipital sites [44]. In addition, it has been also recently proposed that tasks involving a strong endogenous, top-down component are associated with high activity in the beta band [48]. Based on the role of alpha and beta frequencies in visual processing, weaker alpha suppression and decreased beta synchronization—if present in individuals with ASD—would indicate lower attention allocation to visually presented names and attenuated top-down processes in this clinical group.

Finally, recognition of visually presented names requires an interaction between distant posterior and anterior regions: the former is involved in initial processing of visual stimuli [49, 50], the latter—in decision-making whether names are familiar or not (e.g., [51]). Functional communication between these two brain regions is definitely required to successfully accomplish the recognition task, by transferring sensory information from the visual areas to frontal regions involved in goal-directed attention and planning of the appropriate behavioral response [32, 52]. This raises a question whether plausible attentional impairments in the name recognition would be accompanied by a long-range disconnectivity of the autistic brain. Such long-distance under-connectivity (and short-distance over-connectivity) has been proposed by recent explanatory models of autism (for reviews see [53–56]) and a deficit in connections between anterior-posterior regions of the autistic brain was reported in the case of resting-EEG (e.g., [57, 58]). Empirical confirmation of our prediction (on impaired task-related functional connectivity in individuals with ASD) would provide a strong evidence for the connectivity models of ASD; evidence based not on general resting-state activity, but on functional connectivity patterns during the specific behavioral task, i.e., the name recognition.

Therefore, the aim of this EEG study was to investigate electrophysiological correlates of name processing in ASD; in particular, we were interested in possible attention and connectivity deficits during the name recognition task. In order to achieve these goals, self-, close other’s, famous, and unknown names were visually presented to the participants who decided whether each name was familiar or unfamiliar to them. EEG was continuously recorded during this task and analyzed using a range of the following methods. Brain activity was assessed by ERP and event-related desynchronization and event-related synchronization (ERD/S) [59] and functional connectivity—by coherence as a function of time [60] and directed transfer function (DTF) [61, 62]. The latter enables estimation of not only the strength but also the direction of activity flow from one location to another.

Based on evidence discussed earlier, we hypothesized that in our ASD group, the following effects would be present: (i) lack of preferential processing of one’s own name, reflected in similar P300 responses to the self- and close-other’s names, (ii) weaker alpha ERD and weaker beta ERS, and (iii) impaired task-related connectivity between parietal-occipital and frontal brain areas.

## Methods

### Participants

Nineteen young males with ASD and 19 control individuals participated in this study. Control participants were matched one-to-one to individuals with ASD in terms of age, sex, handedness, and IQ-score. Four individuals with ASD were excluded from the analyses due to excessive artifacts in the EEG signal. Consequently, the four control subjects that matched those individuals with ASD were also excluded. Thus, the final size of each group was 15.

Subjects’ IQs were evaluated using the Wechsler Intelligence Scale for Adults - Revised (WAIS-R, PL) [63]. The maximal IQ difference between each individual with ASD and the matched control subject was ± 15 (see Table 1). The maximal age difference between each individual with ASD and the matched control subject was ± 5 months. In the ASD group, the age ranged from 16 years and 7 months to 23 years, the mean age was 19 years and 3 months (SD = 2 years and 4 months). In the control group, the age ranged from 16 years and 11 months to 22 years and 8 months, the mean age was 19 years and 2 months (SD = 2 years and 2 months).

**Table 1:** Demographic and cognitive characteristics of the participants

Subject	Hand	IQ			Subject	Hand	IQ			ADI-R			ADOS	
		Verbal	Perf.	Full			Verbal	Perf.	Full	Social (10)	Com. (8)	RSB (3)	Social (4)	Com. (2)
C1	R	111	126	118	A1	R	124	93	111	16	21	7	5	2
C2	R	139	122	132	A2	R	114	123	118	24	17	9	12	4
C3	R	107	114	110	A3	R	108	122	114	30	26	12	9	6
C4	R	99	93	97	A4	R	100	69	86	25	23	7	3•	3
C5	R	116	126	121	A5	R	119	122	121	25	17	5	9	3
C6	R	111	108	110	A6	R	108	83	97	21	22	8	6	6
C7	R	86	99	91	A7	R	85	95	89	30	20	12	13	4
C8	R	114	112	113	A8	R	101	105	103	21	20	7	6	4
C9	R	130	97	116	A9	R	109	103	106	27	23	12	6	3
C10	R	130	123	128	A10	R	125	107	117	25	22	10	7	3
C11	R	86	93	89	A11	R	96	109	102	25	23	8	8	5
C12	R	120	112	117	A12	R	116	93	106	27	21	11	8	3
C13	L	110	110	110	A13	L	113	112	113	25	20	6	11	4
C14	R	116	117	117	A14	R	104	118	112	26	14	5	11	6
C15	L	122	92	109	A15	L	119	121	122	27	21	8	5	3

Left side: handedness and IQ scores of control participants (C1-C15). Right side: handedness, IQ scores, ADI-R and ADOS scores of individuals with ASD (A1-A15). In all cases, age differences between an individual with ASD and a matched control participant was ≤ 5 months. All subjects were males. (•) marks ADOS score that did not meet the criterion for ASD; nevertheless, this person was included into the ASD group because he had a psychiatric diagnosis of ASD and met all other criteria. Numbers in parentheses indicate cut-offs for ADI-R and ADOS subscales

*Abbreviations*: *Perf.* performance IQ, *Com.* communication, *RSB* repetitive and stereotyped behavior

The clinical diagnosis of ASD subjects was confirmed using standardized tests: the Autism Diagnostic Observation Schedule – ADOS (module 4) [64, 65] and the Autism Diagnostic Interview-Revised – ADI-R [66]. Autistic traits in the control participants, in turn, were controlled by the Autism Spectrum Quotient (AQ) [67]; the range of AQ scores was 11–19 and the mean AQ score (± SD) was 14.9 ± 3.2.

Handedness was confirmed with the Edinburgh Inventory [68]. Subjects had normal or corrected-to-normal vision, and they did not take any medication at the time of experiment. Subjects were financially compensated for their participation.

### Stimuli

Stimuli (first and last names, further referred to as names) were presented visually (white letters against a black background). The size of the stimuli ranged from 2° × 2° to 2° × 6°. The names belonged to four categories: subject’s own name (50 presentations), name of a close-other (50 presentations), name of a famous person (e.g., an actor, 50 presentations), and unknown names (three names, each presented 50 times). Three unknown names were used in order to equalize the probability of behavioral response for unfamiliar (150) and familiar (150) names.

No restriction was placed on the subjects’ choice of the close-other as we wanted to avoid a situation where the pre-defined close-other is not really close to a particular subject [13, 29, 69, 70]. Thus, prior to the experiment, participants were asked to choose a person who was currently (i.e., at the time of our experiment) the most significant to them and describe their relationship briefly. In the ASD group, ten participants chose their parent’s name; two – sibling’s name; two – grandmother’s name; and one – best friend’s name. In the control group, five participants chose their parent’s name; three – sibling’s name; one – grandmother’s name; two – best friend’s name, and four – girlfriend’s name.

In fact, the whole set of stimuli was individually tailored, i.e., for each subject, different famous and unknown names were chosen to match the length of self- and close-other’s names. Stimuli were presented in a pseudo-random order with no more than three names of the same category presented consecutively. Before the experiment, each participant was asked to confirm that he knew the famous name (“What is the profession of this person?”) and did not know the unknown names (“Do you know anybody whose name is … ?”).

### Experimental procedure

Stimuli were displayed in central vision on a 19-inch NEC MultiSync LCD 1990Fx monitor. Presentation® software (Neurobehavioral Systems, Albany, CA, USA) was used for stimuli presentation and response logging. The participants were seated in an acoustically and electrically shielded dark room at a distance of 60 cm from the computer monitor. Subjects performed a speeded two-choice recognition task: unfamiliar vs. familiar (i.e., own-name, close-other’s, and famous names). They responded by pressing one of the two buttons on a Cedrus response pad (RB-830, San Pedro, USA) using the index and the middle fingers of the right hand to press the keys. Key assignment was counterbalanced across subjects.

After reading the instructions displayed on the computer screen, the participants started a trial session in which feedback information was displayed (i.e. “correct”, “incorrect”, or “reaction too slow!”). The experimental session followed immediately after. In each trial, presentation of a fixation point (a white “+” against a black background) for 100 ms was followed by a blank screen for 500 ms, after which a target item (a name) was displayed for 500 ms. Next, the participants were shown a blank screen for 2000 ms, during which they were to give a response. The inter-trial interval varied between 100, 200, and 300 ms. The whole experiment lasted about 15 min.

### EEG recordings

EEG was continuously recorded from 62 scalp sites and two additional electrodes placed on the left and right earlobes. We used a 136-channel amplifier (QuickAmp, Brain Products, Enschede, Netherlands) and the BrainVisionRecorder® software (Brain Products, Munich, Germany). Electrodes were mounted on an elastic cap (ActiCAP, Munich, Germany) and positioned according to the extended 10–20 system. Electrode impedance was kept below 5 kΩ. EEG signal was recorded against the average of all channels calculated by the amplifier hardware. The sampling rate was 500 Hz.

### Behavioral data analysis

Responses were scored as correct if the appropriate key was pressed within 150–2000 ms after the stimulus onset. Pressing the wrong key or pressing no key at all was treated as an incorrect response. Reaction times (RTs) were averaged across correct trials only. For each participant, percentage accuracy and RTs were analyzed only for one unfamiliar name, chosen from the set of all three unknown names. The reason for selecting one unfamiliar name was to match the number of repetitions between four categories of stimuli (for details, see the “EEG data analyses” section).

The selection of unknown names was random and balanced in the sense that, for one participant, we selected the unknown name that was matched to the length of the self-name, for the next participant, we selected the unknown name that was matched to the length of the close-other’s name, etc. This procedure was the same in both groups. Crucially, different unknown names were used for different participants thus it is unlikely that our selection procedure introduced any systematic bias.

RTs and accuracy rates were analyzed using mixed-model ANOVA with group (ASD, control) and category of name (own, close-other, famous, and unknown) as factors. All effects with more than one degree of freedom in the numerator were adjusted for violations of sphericity according to the Greenhouse-Geisser formula [71]. The analyses were conducted in the IBM SPSS Statistics 21 Advanced Model.

### EEG data analyses

#### ERP

The ERP analysis was performed using the BrainVisionAnalyzer® software (Brain Products, Gilching, Germany). Preprocessing steps were analogous to those used in our previous study on name detection in ASD [29] since we aimed to compare the current and the previous P300 findings. First, EEG data were re-referenced to the averaged earlobes and then Butterworth zero phase filters were implemented: high-pass – 0.1 Hz, 12 dB/oct, low-pass – 30 Hz, 12 dB/oct, and notch filter – 50 Hz. Correction of ocular artifacts was then performed using the Independent Component Analysis – ICA [72]. After the decomposition of each data set into maximally statistically independent components, the components representing eye blinks were rejected based on the visual inspection of the component map [73]. Ocular-artifact-free EEG data were obtained by back-projecting the remaining ICA components after they were multiplied using the reduced component-mixing matrix. Next, the EEG was segmented to obtain epochs extending from 200 ms before to 1000 ms after the stimulus onset (baseline correction from −200 to 0 ms). In the automatic artifact rejection, the maximum permitted voltage step per sampling point was 50 μV. In turn, the maximum permitted absolute difference between two values in the segment was 200 μV. The minimum and maximum permitted amplitudes were −200 and 200 μV, respectively, and the lowest permitted activity difference in the 100 ms interval was 0.5 μV.

ERPs for own, close-other’s, famous, and unknown names were computed for correct trials only. ERPs for the unknown category were computed only for one of the unknown names per subject, randomly chosen from the set of all three unknown names. This was done to have similar number of trials for different experimental conditions, thus to avoid problems with different signal-to-noise ratios in different experimental conditions (the higher the number of trials/repetitions, the higher the signal-to-noise ratio). Since we had three unfamiliar names (each presented 50 times, resulting in 150 trials in the unfamiliar condition) and one self-name (50 repetitions), one close-other’s name (50 repetitions), and one famous name (50 repetitions), for each participant one unfamiliar name (50 repetitions) was selected for further analyses.

The mean number of segments used to compute ERPs (in ASD and control groups, respectively) was as follows: own name - 48, 48, close other’s name - 49, 47, famous name - 47, 47, and unknown name - 48, 48. We did not find any significant differences in the number of epochs used to compute ERPs between name categories or between groups. Trials were averaged individually for each electrode site, for each participant, and for each stimulus condition.

For each experimental condition, P300 amplitude was calculated as the mean of values at each time point within the 400–550 ms time window (i.e., the mean amplitude method). This method is less affected by the possibly low signal-to-noise ratio than the peak amplitude method [74]. Mean amplitudes of P300 were analyzed at CPz that is the typically selected electrode location for the analysis of P300 (e.g., [12, 29, 75]).

#### ERD/S, coherence as a function of time, and DTF

ERD/S reflects relative changes of the EEG spectral power recorded after the stimulus onset in comparison to a reference period registered before the stimulus presentation [59]. Quantification of ERD/S was performed in time and frequency domains and was based on a method similar to the event-related spectral perturbation (ERPS) proposed by Makeig [76].

Coherence is a measure of synchronization between two signals based mainly on phase consistency. Coherence indicates the level of synchronization in activity between different neural populations, where high coherence reflects greater functional integration due to either cortico-cortical or cortical-subcortical-cortical connections [60]. In order to obtain its time course, we estimated coherence in a way similar to event-related coherence [77]. This is a method for the analysis of coherence between electrodes as a function of time (see Additional file 1 for the detailed description), and it generates coherence values for the entire time-frequency spectrum, allowing the analysis of coherence related to particular events in time, such as the presentation of visual stimuli.

DTF, in turn, measures causal interactions in the frequency domain between two EEG channels, with respect to connections between all other available channels. DTF enables estimation of a strength and direction of activity flow from one location to another [61, 62]. DTF is defined within the framework of the Mulivariate Autoregressive Model – MVAR [61]. In comparison to coherence, a great advantage of the MVAR approach is that it accounts for the whole multivariate set of signals, so the analysis is not performed separately for every pair of signals (which is the case for coherence), thus eliminating the problem of a presence of common sources in the set of signals [78]. Detailed description of ERD/S, coherence, and DTF calculations is provided in Additional file 1.

The EEG data preprocessing was as follows. EEG data were re-referenced to the averaged earlobes and then down-sampled to 250 Hz. Next, the signals were segmented into trials with respect to the onset of the fixation point. Trials with amplitudes exceeding ±125 μV were removed from further analysis. Accepted trials were passed to a third-order Butterworth bandpass filter in the frequency range of 3.0–32 Hz.

Then EEG signals were decomposed by means of ICA [72], implemented into EEGLab using extended Infomax. All components identified as a source of eye movements or muscle artifacts were removed, and then the remaining ICA components were used to reconstruct the signal in the original electrode space. Next, a 1500-ms segment was extracted from each trial in each experimental condition, with respect to the onset of the blank screen epoch that followed the presentation of the fixation point. In the case of DTF, the second step of artifact rejection was omitted because ICA disturbs the fitting of the MVAR model to EEG data [79].

The reasons are as follows. The MVAR model assumes that the amplitude of signal at a given channel and time sample can be described as a linear combination of previous samples derived from itself or from other channels with an added unpredictable random component (noise). According to this model, all channels of the multivariate signal may be more or less correlated but they are linearly independent. The procedure of the artifact rejection performed by ICA consists of three major steps: (i) decomposition of the original signal by ICA; (ii) removal the ICA components identified as sources of artifacts; and (iii) reconstruction of EEG signal from the remaining ICA components. As one can see, the steps (ii) and (iii) lead to removal from each channel of initial signal the same activity recognized as undesirable distribution. Thus, steps (ii) and (iii) make the channels of reconstructed, artifacts-free multivariate signal linearly dependent. However, the condition of the linear independence of the channels must be absolutely satisfied if he MVAR model has to be fitted to data.

The number of EEG channels that could be used for estimation of the MVAR model was restricted by the number of available samples. In our study, the amount of the measured EEG data allowed the MVAR model to be fitted for up to 17 electrodes. Thus, 17 electrodes from the 62 available sensors were selected within our two regions of interest: frontal (F7, F5, F3, F1, FZ, F2, F4, F6, and F8) and parieto-occipital (P7, PO7, O1, OZ, O2, PO8, P8, and Iz). The criteria for selection were as follows: (i) electrodes had to be evenly distributed within each region of interest; (ii) in the case of the frontal region, they had to be at some distance away from the most anterior electrode sites that are typically strongly influenced by eye-movements artifacts (in the case of DTF calculations, ICA-based artifact rejection had to be omitted); and (iii) the number of electrodes located within the left and right hemisphere had be the same (we had no hypothesis regarding lateralization). ERD/S, coherence, and DTF were calculated for the same set of electrodes.

ERD/S was analyzed at each of the 17 electrodes in the following frequency ranges (within the theta, alpha, and beta bands, respectively): 4–8 Hz in 100–250 ms time window, 10–13 Hz in 350–800 ms time window and 13–18 Hz in 100–250 ms time window. Selection of time windows and frequency ranges was guided by results of ERD/S collapsed across groups and conditions (see Additional file 2, Figure A1) [80].

Coherence was statistically analyzed for selected pairs of electrodes. In order to avoid the double-dipping problem [81], such selection has to be orthogonal to potential differences between groups or experimental conditions. To this end, we (i) collapsed the EEG signal across the two groups (ASD, controls) and across the four name categories (self, close-other's, famous, and unknown); (ii) noticed that local (i.e., within-region) effects were very weak whereas between-regions effects (i.e., long-range connections) were clearly visible (see Additional file 2, Figure A2); (ii) calculated the average coherence values for the theta, alpha, and beta bands based on all posterior-anterior pairs of electrodes; and finally, (iii) selected pairs of electrodes in which the coherence values were higher than the average calculated for a given frequency band.

In this way, we selected 13, 11, and 21 pairs of electrodes for the analyses of coherence in the theta, alpha, and beta bands, respectively, and the following statistical analyses were run only on these pairs of electrodes. Our selection procedure is independent from our main analysis; the selection was based on the collapsed data from both groups and all conditions, whereas the analysis compared the groups/conditions between each other. Thus, without introducing any bias [81], we could limit the number of electrodes pairs in our analysis and lower the risk of type II error due to the correction for multiple comparisons.

Selection of time windows and frequency ranges was also based on coherence results collapsed across groups and conditions (see Additional file 2, Figure A2) [80]. Coherence was analyzed in the following frequency ranges (within the theta, alpha, and beta bands, respectively): 4–8 Hz in 250–650 ms time window, 10–13 Hz in 500–800 ms time window, and 21–28 Hz in 250–750 ms time window.

DTFs were calculated for three subsequent time windows: 0–200, 200–400, and 400–600 ms. DTF calculations were restricted to frequencies within the beta band: low (13–18 Hz) and high (18–30 Hz) as moderate and long-distance cortical connections are based mainly on beta oscillations [82, 83]. This is also consistent with the evidence pointing to the role of beta oscillations in attentional processes in the visual domain [44, 84–87].

Selection of connections for statistical analyses was based on a procedure that was orthogonal to potential group differences [81] and was done on the basis of the DTF averaged for the control and ASD groups (see Additional file 2, Figures A3, A4, and A5). Similarly to the selection procedure used for the coherence, connections with DTF values higher than the average were further statistically compared in the two groups. The number of connections was 56, 64, and 45, for the 0–200, 200–400, and 400–600 ms time-window, respectively.

#### Statistical analyses

Amplitudes of P300, ERD/S (for single electrodes) and coherence (for single pairs of electrodes) were analyzed using mixed-model ANOVA with group (ASD, control) and category of name (own, close-other, famous, and unknown) as factors. Bonferroni correction for multiple comparisons was applied to the post-hoc analyses. All effects with more than one degree of freedom in the numerator were adjusted for violations of sphericity according to the Greenhouse-Geisser formula [71]. The analyses were conducted in IBM SPSS Statistics 21 (Advanced Model). The statistical significance of DTF differences between the ASD and control groups (for each connection) was verified by a two-sided Wilcoxon rank sum test using MATLAB statistical toolbox. Finally, the multiple comparisons problem arising in the analyses of ERD/S, coherence, and DTF was addressed by applying the false discovery rate (FDR) correction to the obtained *p* values [88]. The maximum FDR level (*q* value) was set to 5 %.

## Results

### Behavioral results

The mean percent of correct responses and mean RTs are presented in Table 2. Mixed-model ANOVA revealed that the main factor of group and category of name as well as their interaction were non-significant neither for RTs (group: F(1,28) = 2.010, *p* = 0.168; name: F(3,26) = 1.167, *p* = 0.318; group x name: F(3,84) = 0.757, *p* = 0.471) nor accuracy rates (group: F(1,28) = 1.974, *p* = 0.171; name: F(3,26) = 2.014, *p* = 0.096; group x name: F(1,28) = 2.322, *p* = 0.125).

**Table 2:** Mean reaction times (RTs) ± SD and accuracy rates in control participants and individuals with ASD for each category of name

	Control group				ASD group			
	Self	Close-other	Famous	Unknown	Self	Close-other	Famous	Unknown
RTs (ms)	493 ± 99	524 ± 114	583 ± 105	567 ± 139	595 ± 237	636 ± 254	669 ± 267	659 ± 263
Accuracy (%)	99 ± 01	98 ± 04	94 ± 06	98 ± 03	98 ± 02	95 ± 04	94 ± 04	95 ± 05

### ERP

**Fig. 1:**
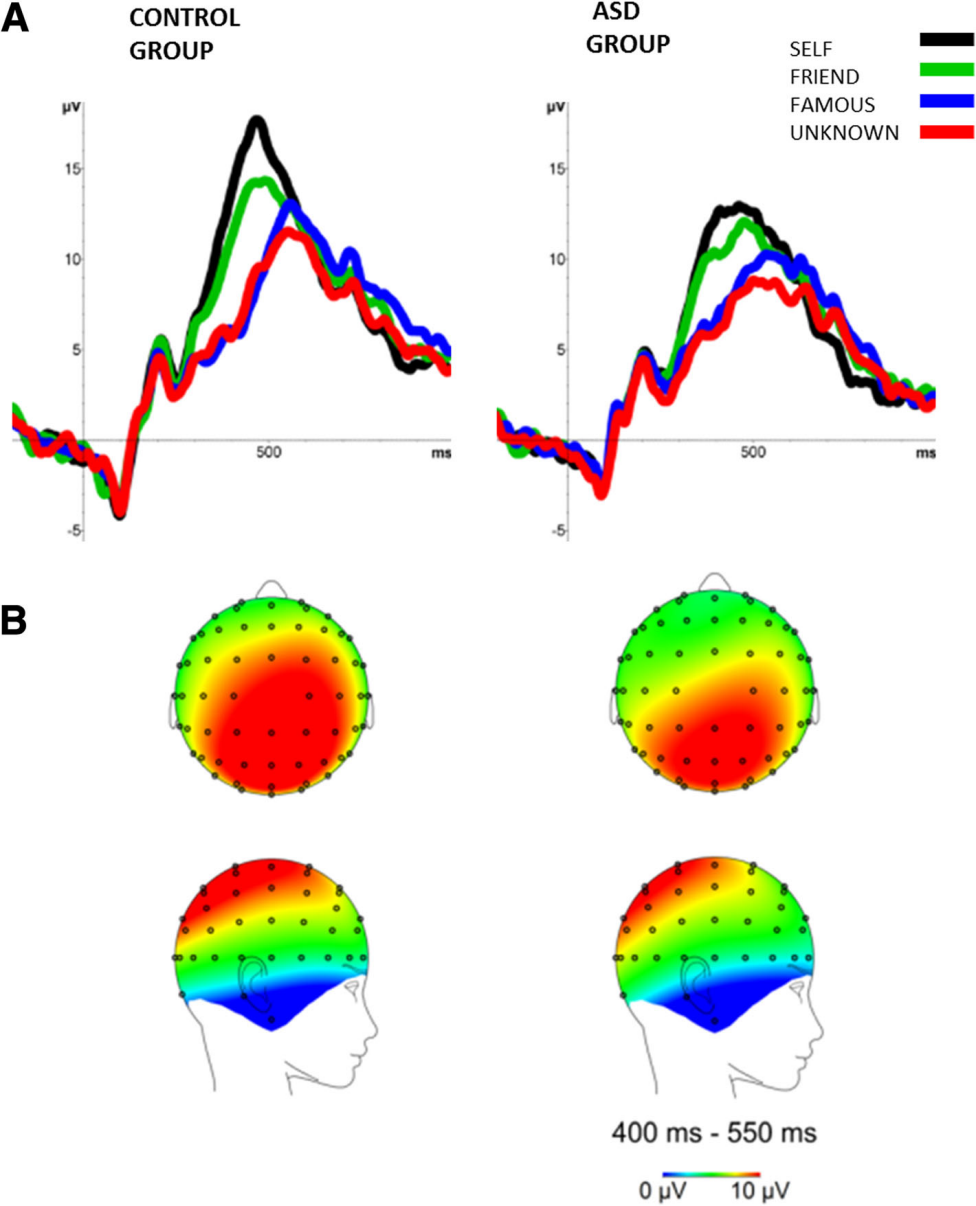
Grand average ERPs at CPz in the control group (**a** - *left panel*) and in the group of individuals with ASD (**a** - *right panel*). Topographical distribution of P300 in the control group (**b** – *left panel*) and in the group of individuals with ASD (**b** – *right panel*)

Grand average ERPs for the ASD and control groups are presented in Fig. 1. Mixed-model ANOVA revealed a significant main effect of the category of name (F(3,84) = 31.223, *p* < 0.001, η_p_^2^ = 0.527), and an interaction between the group and name-category factors (F(3,84) = 3.170, *p* = 0.04, η_p_^2^ = 0.102). Post-hoc tests indicated larger amplitudes to one’s own name than to close-other’s (*p* = 0.030), famous (*p* < 0.001), and unknown (*p* < 0.001) names. Moreover, P300 amplitudes for close-other’s name were larger than for famous (*p* < 0.001) and unknown name (*p* < 0.001).

Post-hoc comparisons for the interaction showed that in the control group P300 to one’s own name was higher than P300s to all other names (close-other’s: *p* = 0.008, famous: *p* < 0.001, unknown: *p* < 0.001), and also that P300 amplitudes to close-other’s name were higher than P300 to famous (*p* = 0.001) and unknown (*p* = 0.005) names. In the ASD group, however, P300 response to one’s own name did not differ from P300 response to close-other’s name (*p* > 0.9), but it was significantly higher than P300 amplitudes to famous (*p* = .015) and unknown (*p* = 0.003) names.

### ERD/S

Results of mixed-model ANOVAs indicated differences between ASD and control group for the alpha, theta, and beta frequency ranges. All those results are presented in Additional files 3 and 4 (Figure A6 and Table A1, respectively). After FDR correction for multiple comparisons, no effects remained significant.

### Coherence

Mixed-model ANOVAs revealed between-group differences (i.e., a significant main factor of ‘group’) for alpha, theta, and beta frequency ranges. All those results are presented in Additional files 3 and 4 (Figure A7 and Table A2, respectively). The category of name factor as well as its interactions with the group factor were non-significant in all ANOVAs.

**Fig. 2:**
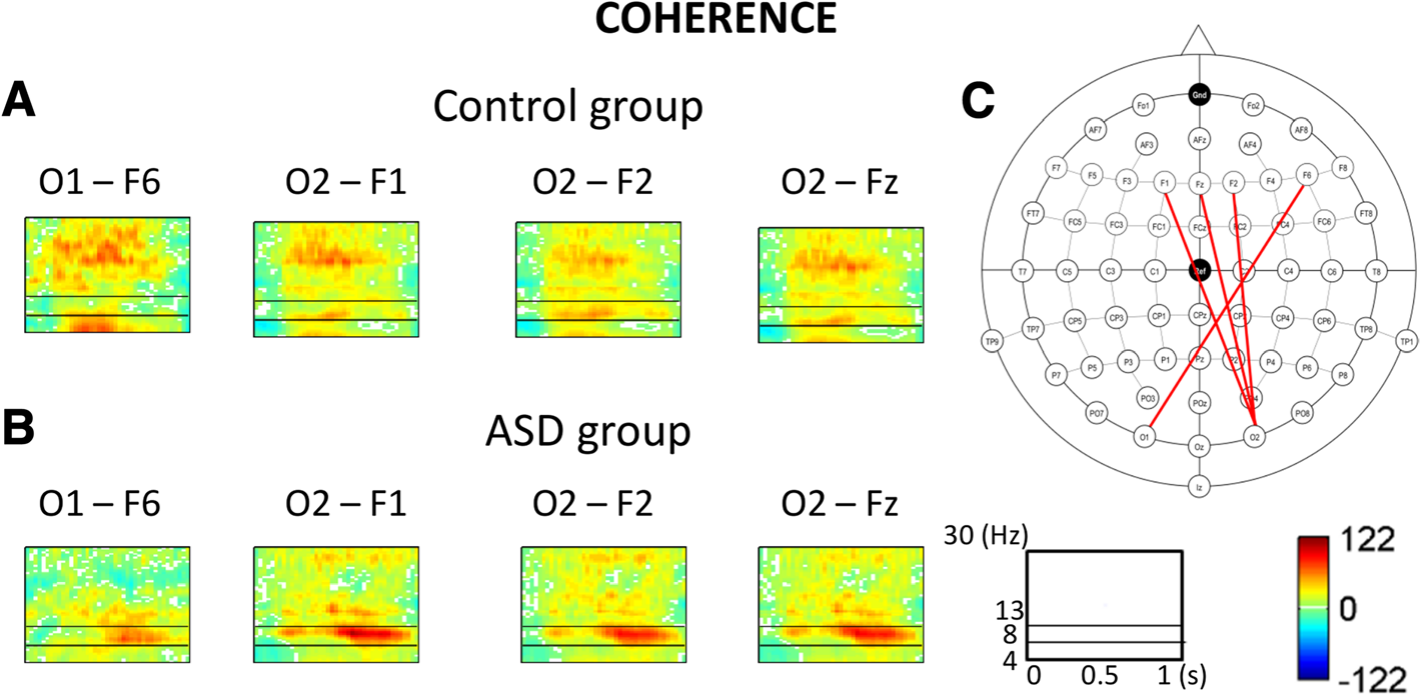
Time course of coherence averaged across all categories of names in the control group (**a** – *upper panel*) and in the ASD group (**b** – *lower panel*). *Red lines* in the scheme of the extended 10–20 system indicate significantly enhanced coherence (i.e. stronger connections) within the beta band in the control group in comparison to the ASD group (**c**). F – frontal electrodes; O – occipital electrodes. Odd numbers (e.g., F1) - electrodes located over the left side of the head; even numbers (e.g., F6) - electrodes located over the right side of the head; “z” (e.g. Fz) - electrodes located at the midline

After FDR correction for multiple comparisons, decreased coherence in the ASD group in reference to the control group was found within the beta band for the following pairs of electrodes: Fz-O2 (*p* = 0.009), F1-O2 (*p* = 0.024), F2-O2 (*p* = 0.024), and F6-O1 (*p* = 0.027). Those results are presented in Fig. 2.

### Directed transfer function

In the ASD group, under-connectivity (i.e., lower DTF values) was found for long-range connections going from parietal-occipital to frontal sites, accompanied by under-connectivity within the frontal region and over-connectivity within parietal-occipital region. Results that reached the significance level (*p* < 0.05, uncorrected) are presented in Additional files 3 and 4 (Figure A8 and Table A3, respectively).

**Fig. 3:**
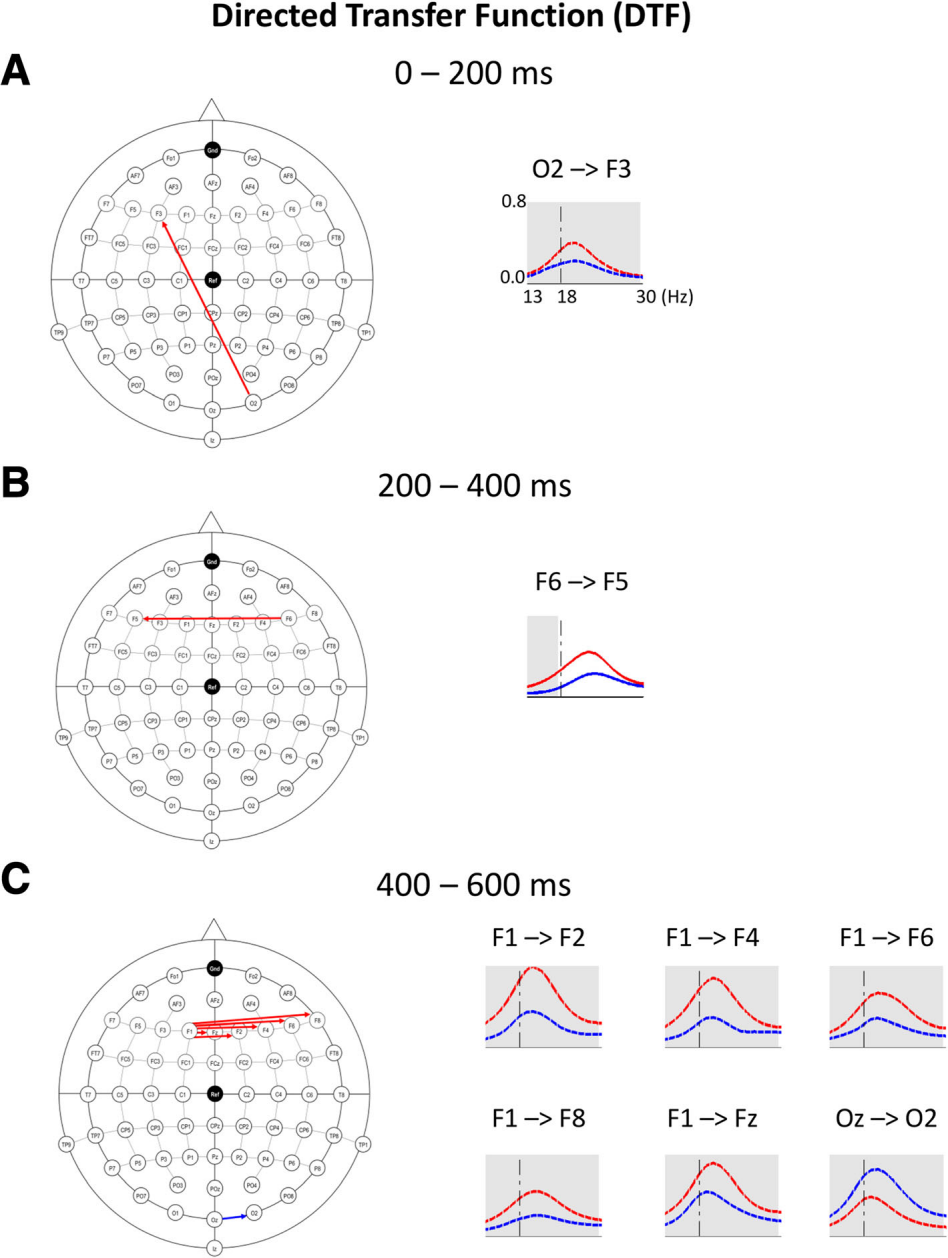
Directed transfer function (DTF) for the three consecutive time windows: 0–200 ms (**a**), 200–400 ms (**b**), and 400–600 ms (**c**). Please note that 0 ms corresponds to the stimulus onset. DTF graphs (*right panel*) present results in the control group (*red line*) and in the ASD group (*blue line*). *Gray-colored rectangles* indicate significant (FDR-corrected) between-group differences. These differences are illustrated also on the scheme of the extended 10–20 system (*left panel*). *Red arrows* represent connections that are significantly stronger in the control group than in the ASD group, blue arrow – connections significantly stronger in the ASD group than in the control group. F – frontal electrodes; O – occipital electrodes. Odd numbers (e.g., F1) - electrodes located over the left side of the head; even numbers (e.g., F6) - electrodes located over the right side of the head; “z” (e.g. Fz) - electrodes located at the midline

After FDR correction for multiple comparisons, lower DTF values in the ASD group, when compared to the control group, were found for the long-range posterior-to-anterior connection: O2 → F3 (0–200 ms, 18–30 Hz, *p* = 0.03), and for a number of connections within the frontal region: F6 → F5 (200–400 ms, 13–18 Hz, *p* = 0.02), F1 → Fz (400–600 ms, 18–30 Hz, *p* = 0.01), F1 → F2 (400–600 ms, 13–18 Hz, *p* < 0.001; 18–30 Hz, *p* < 0.001), F1 → F4 (400–600 ms, 13–18 Hz, *p* < 0.001; 18–30 Hz, *p* < 0.001), F1 → F6 (400–600 ms, 13–18 Hz, *p* = 0.02; 18–30 Hz, *p* = 0.01), and F1 → F8 (400–600 ms, 13–18 Hz, *p* < 0.001; 18–30 Hz, *p* < 0.001). Moreover, higher DTF value in the ASD group than in the control group was observed for the local occipital connection: Oz → O2 (400–600 ms, 18–30 Hz, *p* = 0.01). FDR-corrected DTF results are presented in Fig. 3.

## Discussion

In this EEG study, we investigated patterns of brain activity and functional connectivity associated with names processing in individuals with ASD. In the control group, ERP results showed enhanced P300 to one’s own name in comparison to all other names, whereas in the ASD group P300 to one’s own name and close-other’s name did not differ. The latter suggests equivalent attention allocation for one’s own and close-other’s names in the ASD group. An analogous pattern of P300 findings in individuals with ASD was previously reported in the case of name detection (see the Introduction and [29]). This is of particular interest since recognition of names in the present study involves mainly goal-directed attention, whereas detection of names (no discrimination required) involves mainly bottom-up attention. Despite the fact that the two experiments differed with respect to attentional requirements, both detection and recognition of stimuli did not lead to neural differentiation between one’s own and the close-other’s names in the ASD group. Apparently, enhanced top-down attention during the name recognition task did not improve the impaired—on the neural level—discrimination of self-name from the close-other’s name in individuals with ASD. In general, this finding may be also related to the concept of disturbed neural self-representation in individuals with ASD [89–92].

While the inter-group differences in ERPs were influenced by the category of name, two measures of task-related connectivity within the beta band showed inter-group effects common for all names. Coherence in the beta band was significantly higher in the control group than in the ASD group for distant inter- and intra-hemispheric connections, i.e., between posterior (parietal, occipital) and anterior (frontal) regions. DTF analysis revealed weaker connectivity between anterior and posterior brain regions in the ASD group compared to the control group and—importantly—this method indicated the directionality of these weaker connections. Disruption of functional connectivity observed in individuals with ASD was present mainly in the connections from parietal-occipital to the frontal electrode sites. In addition, strong connections within the frontal region were present in the control group, whereas in the ASD group such over-connectivity was present in the parietal-occipital region. In general, the crucial role of beta oscillations in attentional processes, especially in the visual domain, is well-documented (e.g., [44, 84–87]). Moreover, communication within the attentional network proceeds mainly via long-range synchronization in the beta band [45]. Therefore, impaired long-range connectivity in the beta frequency range, observed in our group of participants with ASD, may be linked to disturbed attentional processes and suggests some dysfunction of the attentional network involved in name recognition.

Thus, in the ASD group, we found no the self-preference effect (i.e., similar P300 to one’s own and the close-other’s name), disruption of functional long-range (anterior-posterior) connectivity, decreased short-range connections in the frontal region, and increased short-range connections in the parietal-occipital region. In general, our results are in line with recent EEG studies investigating connectivity patterns in individuals with ASD [55, 57, 93–96]. Specifically, attenuated long-range communication between the frontal lobes and posterior parts of the autistic brain was reported in numerous studies (e.g., [57, 58, 93, 96]). Increased coherence, in turn, was found within frontal, temporal, and occipital sites in the ASD group [58]. However, some studies also reported lower coherence values for local connections, e.g., between frontal electrodes in the right hemisphere [97].

At this point, it should be noted that the majority of previous ASD studies investigated connectivity patterns in the resting states, with eyes-open [98] or eyes-closed [57, 58, 93] and even during sleep [53], while our results refer to active processing of social stimuli. Thus, it is a matter of debate whether resting-state connectivity and task-related connectivity shows similar or rather dissimilar patterns. Nevertheless, the DTF and coherence results of our study corroborate the notion of a double dissociation of connectivity patterns in individuals with ASD, i.e., a lack of long-range connections, with the most prominent deficit in the fronto-occipital connections and increased short-range connections. Common effects found in different cognitive states—active vs. rest—provide converging evidence of connectivity disruption in ASD.

Importantly, long-range under-connectivity in ASD was reported only in few EEG and MEG studies in which participants were involved in cognitive tasks. Using wavelet transform coherence, Catarino et al. [99] showed a widespread and consistent reduction in interhemispheric connectivity in the ASD group compared to the control group during visual perception and categorization of social and inanimate stimuli. These effects were found across the entire time-frequency spectrum, though they were most pronounced at frequencies lower than 13 Hz. In a MEG study, disruption of long-range phase synchronization among frontal, parietal, and occipital areas was found in high-functioning children with ASD during the performance of executive function tasks [100]. In addition, a significant prefrontal synchronization was found in control compared to ASD participants mostly at the frequency range between 16 and 34 Hz. These effects are in line with our results showing significantly stronger coherence in high beta (21–28 Hz) in the frontal region and enhanced DTF at the frequency range of 13–30 Hz in the control group compared to the ASD group.

Interestingly, our coherence and DTF results both for long- and short-range connectivity (described and discussed above) match also findings of functional connectivity magnetic resonance imaging (fcMRI) studies in ASD [94, 101, 102]. Specifically, those studies showed frontal-posterior under-connectivity [43] and over-connectivity in the posterior occipital cortex alongside local hypo-connectivity in medial prefrontal regions in the autistic brain [94, 102] (see our DTF findings, Fig. 3).

It is worth to note that present findings revealed a dissociation between behavioral and neural results; while significant between-groups and/or between-conditions differences were found in ERP, coherence, and DTF analyses, no significant effects were present in the behavioral data. That is, recognition rates for all names were similarly high in individuals with ASD and control participants for all names and no between-group difference in RTs reached statistical significance. Such dissociation between the neural and behavioral data is not unusual [29, 99, 103, 104]. In general, similar levels of behavioral functioning in the ASD group and control participants may result from compensatory strategies applied by individuals with ASD (e.g., [104]). This compensation seems especially plausible in the present study as only high-functioning ASD participants were tested.

We would like to briefly mention some ERD/S, coherence and DTF results that did not survive FDR corrections. Similarly to FDR-corrected findings of our study, some of these effects also pointed to impairments in attentional processes involved in the name recognition in individuals with ASD. In the ASD group in reference to the control group, we found weaker de-synchronization and higher coherence within the alpha band in the occipital region. Alpha suppression typically occurs when attention is directed towards external stimuli [37] and following visual stimulation can be observed in the corresponding sensory area, i.e., the occipital region [38]. In the present study, this was the case in the control group but not in the ASD group. We observed also decreased beta synchronization in individuals with ASD. This finding, in turn, may be related to either attenuated top-down processes or stronger involvement of bottom-up processes [48]. Moreover, in the ASD group, synchronization within the theta band was weaker and coherence was lower in comparison to the control group. Although the theta band is most commonly related to memory processes [105], it has also been linked to emotional arousal [37]. Previous studies showed that theta power increase was observed in response to emotional compared to neutral stimuli, and the earliest discrimination of the emotional content of stimuli occurred in the lower theta range during the first 600 ms after stimulus onset [106]. Similarly, in the present study, inter-group differences appeared in the lower theta band, within the similar time range. Therefore, reported effects in the theta band may indicate that names are treated as stimuli less emotional by individuals with ASD. Finally, the only connection from the parieto-occipital to the frontal region that was stronger in the ASD group than in the control group was found in the 200–400 ms time window. This may indicate some delay in the activity flow from occipital to frontal location in individuals with ASD because in the control participants such enhanced connections were present in the earliest time window (0–200 ms).

Finally, we would like to comment on the limitations of the present study. First of all, the study is confined by a small sample size. Thus, reported results should be treated with caution and they need to be further investigated in larger groups of individuals with ASD in order to increase effect sizes and enhance statistical power. In addition, higher (than 50) number of experimental trials would also be beneficial; with such an increased number of repetitions some effects related to the name category (that were non-significant in our ERD/S, coherence, and DTF analyses) may be detected. Moreover, in the current study names were presented in the visual modality. However, undoubtedly spoken names are more “ecologically valid” (spoken versions of one’s own name and other names are more often encountered in everyday life) and the auditory version is more adequate in the context of social communication, i.e. the domain in which ASD individuals have difficulties. Thus, inter-group differences observed for visually presented names may be underestimated/attenuated in comparison to effects present for aurally presented names, and some effects may even be missing. This notion is strongly supported by our earlier fMRI study on name recognition [69]. Visually presented names generally resulted in weaker activations than aurally presented names and some activations associated with self- and close-other’s name were present only for acoustic presentations. In addition, we tested high-functioning adolescents and young adults with ASD. Studies on younger subjects (children) and severely affected individuals could shed more light on the issue of name processing in ASD. However, passive listening to aurally presented names could be more adequate for these groups and we theorize that such studies would reveal even more pronounced impairments associated with name processing than presented here. Future studies are needed to validate this prediction.

## Conclusions

The results of this study demonstrated altered patterns of brain activity and task-related functional connectivity during recognition of visually presented names in individuals with ASD. Thus, the current study provides novel evidence on the neural underpinnings of name-processing in autism; this important aspect of social cognition has been largely overlooked in the previous neuroscientific literature on ASD. Using advanced methods of the EEG data analyses, we could make an effective case further supporting that ASD is related to augmented short-range functional connectivity in the sensory brain regions and attenuated long-range connectivity between the sensory and higher order associative regions (e.g., [96]). This pattern of neural architecture is highly consistent with the behavioral and clinical data showing that people with autism often have problems with integration of separate perceptual features into a single, coherent mental object (e.g., [2]). These converging neuro-cognitive-clinical observations suggest that the effective therapy of ASD symptoms could use existing capabilities of the autistic mind, such as the privileged processing of perceptual parts and details, in order to improve the limitations of the autistic mind, related to the conceptual integration and context-dependent cognitive flexibility.

## Additional files

Additional file 1: Description of ERD/S, coherence, and DTF calculations. (DOCX 133 kb) Additional file 2: Figures illustrating ERD/S, coherence, and DTF averaged across groups and experimental conditions. (DOCX 1165 kb) Additional file 3: Figures illustrating complete results of ERD/S, coherence, and DTF calculations. (DOCX 3256 kb) Additional file 4: Tables with ERD/S, coherence, and DTF results that did not retain the significance after FDR corrections. (DOCX 45 kb)

## Abbreviations

ASD: Autism spectrum disorder
DTF: Directed transfer function
ERD/S: Event-related desynchronization and synchronization
ERP: Event-related potential
FDR: False discovery rate
