# Supplementary material for: Name recognition in autism: EEG evidence of altered patterns of brain activity and connectivity
Source: Mol Autism. 2016 Sep 6;7(1):38. doi: 10.1186/s13229-016-0102-z (PMC5012044; doi:10.1186/s13229-016-0102-z)
Supplement: Additional file 1: — Description of ERD/S, coherence, and DTF calculations. (DOCX 133 kb) [file 13229_2016_102_MOESM1_ESM.docx]

**Calculations of ERD/S, coherence, and DTF**

***ERD/S***

The formula of ERD/S quantity is equivalent to Event-Related Spectral Perturbation – ERSP [1] and represents the relative spectral power changes in time-frequency domain for a given subject and condition at each electrode site.

|  | (1a) |
| --- | --- |
| $\bar{P}^{post}(f,t)=\frac{\sum_{i=1}^{N} P_{i}^{post}(f,t)}{N},$ | (1b) |
| $\bar{P}^{r}(f)=\frac{\sum_{t=1}^{N_{s}} \sum_{i=1}^{N} P_{i}^{pre}(f,t)}{N\cdot N_{s}}$ | (1c) |

where: $P_{i}^{pre}(f,t)$ and $P_{i}^{post}(f,t)$ represents the power of EEG signal at frequency *f* and time *t* in the *i*-th experimental trial in pre-stimulus baseline and in epoch locked to the stimulus onset, respectively, *N* is the number of experimental repetitions, *N*_s_ is the number of samples in the epoch preceding the stimulus (baseline).

We calculated ERD/S by averaging EEG power within selected frequency band Δ*f* and time range Δ*t*:

|  | (2) |
| --- | --- |

We estimated the EEG power in time and frequency domain required by formula (1) by means of Continuous Wavelet Transform (CWT) as follows. For a given subject and experimental condition, each signal $x_{i}^{c}(k,t)$ was convoluted with the complex Morlet’s wavelet $w(t,f_{0})$[2]:

| $w(t,f_{0})=A\exp\left(-t^{2}/2\sigma_{t}^{2}\right)\exp\left(2\pi if_{0}t\right)$ | (3) |
| --- | --- |

and $\sigma_{f}=\frac{1}{2\pi i\sigma_{t}}$.

Wavelets were normalized to have total energy equal to 1. The wavelet family was characterized by a constant ratio $\frac{f_{0}}{\sigma_{f}}=7$ with$f_{0}$ ranging from 4 to 30 Hz in steps of 0.2 Hz. The squared norm of the convolution of the signal $x_{i}^{c}(k,t)$ with the wavelet family resulted in the time-frequency distribution of the energy in experimental condition *c* at *i*-th trial and channel *k*, denoted as$P_{i,k}^{c}(f,t)$.

Next, we estimated ERD/S quantitatively. The $P_{i,k}^{c}(f,t)$ distributions were averaged over trials and the results were marked as $P_{k}^{c}(f,t)$. From the $P_{k}^{c}(f,t)$ we selected 500 ms long epoch preceding the stimulus presentation, which represents the baseline EEG activity $P_{k}^{c,pre}(f,t)$ and 1000 ms long epoch following the stimulus $P_{k}^{post}(f,t)$. After calculating the mean power for each frequency band in baseline epoch, denoted as$\bar{P}_{k}^{c,pre}(f,t)$, we substituted $P_{k}^{post}(f,t)$ and $\bar{P}_{k}^{c,pre}(f,t)$ to eq. (1a). Finally, we obtained the $ERDS_{k}^{c}\left(f,t\right)$ quantity, which represents the relative spectral power changes in time-frequency domain for a given subject and condition *c* at channel *k*.

To verify statistical significance of results, we tested (depending on the experimental condition) $ERDS_{k}^{c}\left(f,t\right)$ within selected frequency band Δ*f* and time range Δ*t*, which we denoted respectively as $ERDS_{k}^{c}\left(\Delta f,\Delta t\right)$ and $ERDS_{k}\left(\Delta f,\Delta t\right)$.

***Coherence***

Coherence is a measure of synchronization between two signals based mainly on phase consistency. We used the method of coherence estimation proposed by Challis and Kitney [3]. Let $X_{i}\left(f\right)$ and $Y_{i}\left(f\right)$ denote the complex Discrete Fourier Transform of the signals $x_{i}(t)$ and $y_{i}(t)$ in *i*-th experimental repetition. Then the power spectra averaged over trials (marked respectively $S_{x}\left(f\right)$, $S_{y}\left(f\right)$) and cross spectrum $S_{xy}\left(f\right)$ of the signals $x_{i}(t)$ and $y_{i}(t)$ were calculated according to the equation (4a)-(4b):

| $S_{x}\left(f\right)=\frac{1}{N}\sum_{i=1}^{N} \left\vert X_{i}\left(f\right)\right\vert^{2}$ | $S_{y}\left(f\right)=\frac{1}{N}\sum_{i=1}^{N} \left\vert Y_{i}\left(f\right)\right\vert^{2}$ | (4a) |
| --- | --- | --- |
| $S_{xy}\left(f\right)=\frac{1}{N}\sum_{i=1}^{N} X_{i}\left(f,t_{k}\right)\cdot Y_{i}^{*}\left(f,t_{k}\right)$ | | (4b) |

where *N* is the number of experimental trials. The coherence $Coh_{xy}\left(f\right)$ between signal $x(t)$and $y(t)$ is defined as the cross spectrum $S_{xy}\left(f\right)$ normalized by the power spectra $S_{x}\left(f\right)$ and $S_{y}\left(f\right)$:

| $Coh_{xy}\left(f\right)=\frac{S_{xy}(f)}{\sqrt{S_{x}(f)\cdot S_{y}(f)}}$ | (5a) |
| --- | --- |

and its magnitude is equal to:

| $C_{xy}\left(f\right)=\frac{\left\vert S_{xy}(f)\right\vert}{\sqrt{S_{x}(f)\cdot S_{y}(f)}}$ | (5b) |
| --- | --- |

However, coherence calculated in this way is a function of frequency only. In order to obtain its time course, we estimated it in a way similar to Event-Related Coherence [4]. The following procedure was applied. Each signal $x_{i}^{c}(k,t)$ was divided into 200 ms segments and overlapped in 180 ms, denoted as $x_{i}^{c}(k,n)$, where *n* is the segment number. The signal from selected segments was multiplied by Tukey window and extended using zeros padding to obtain a 1 Hz resolution in Discrete Fourier Transform. Then the signals from the given segment and each pair among 17 channels were used to estimate the magnitude of the coherence according to equation (5b). The results were marked as $C_{k,l}^{c}\left(f,n\right)$, where *k* and *l* are channel numbers and *c* is the experimental condition. Next, for the segments starting with the stimulus onset we calculated the Event Related Coherence between channels *k* and *l* according to the formula:

| $ERC_{k,l}^{c}\left(f,n\right)=\frac{C_{k,l}^{c}\left(f,n\right)-C_{k,l}^{c}\left(f\right)}{C_{k,l}^{c}\left(f\right)}$ | (6) |
| --- | --- |

where $C_{k,l}^{c}\left(f\right)$ is the magnitude coherence averaged for each frequency bands over segments that were whole in pre-stimulus epoch.

In order to verify the statistical significance of effects, we applied the magnitude coherence averaged over selected frequency band $\Delta f$ and time windows $\Delta n$ to $ERC_{k,l}^{c}\left(f,n\right)$ and $ERC_{k,l}\left(f,n\right)$ estimators. We denoted this modified Event-Related Coherence as $ERC_{k,l}^{c}\left(\Delta f,\Delta n\right)$ and $ERC_{k,l}\left(\Delta f,\Delta n\right)$.

***Directed transfer function***

DTF measures the causal interactions in the frequency domain between two EEG channels, in respect to the connection between all other available channels, and indicates the directionality of such interactions. DTF is defined in the framework of the Multivariate Autoregressive Model – MVAR [5]. According to this model, the *i-*th sample in the *k*-th channel$x(k,i)$ can be expressed as a weighted sum of *p*-previous samples and unpredictable random element $\varepsilon(k,i)$:

| $\left[\begin{matrix} x(1,i) \\ \ldots\\ x(K,i) \end{matrix}\right]=\left[\begin{matrix} a_{11}^{1} & ... & a_{1K}^{1} \\ \ldots& \ldots& \ldots\\ a_{K1}^{1} & \ldots& a_{KK}^{1} \end{matrix}\cdot\right]\cdot\left[\begin{matrix} x(1,i-1) \\ \ldots\\ x(K,i-1) \end{matrix}\right]+\ldots+\left[\begin{matrix} a_{11}^{p} & ... & a_{1K}^{p} \\ \ldots& \ldots& \ldots\\ a_{K1}^{p} & \ldots& a_{KK}^{p} \end{matrix}\cdot\right]\cdot\left[\begin{matrix} x(1,i-p) \\ \ldots\\ x(K,i-p) \end{matrix}\right]+\left[\begin{matrix} \varepsilon(1,i) \\ \ldots\\ \varepsilon(K,i) \end{matrix}\right]$ | (7) |
| --- | --- |

where: *K* – is the number of channels, *p* is the model order, and $a_{lk}^{p}$ - is the model coefficient, that describes the contribution of the sample $x(k,i-p)$ into sample$x(l,i)$. The equation (7) can be re-written to the vector-matrix form as follows:

|  | (8) |
| --- | --- |

where $\vec{x}(i)\in R^{K\times1}$ denotes the vector of samples at time point *i* and $\mathbf{A}_{j}\in R^{K\times K}$ is the matrix of model coefficients.

After transformation of the model from time domain (eq. (8)) to frequency domain, the MVAR model can be expressed in the following form:

|  | (9) |
| --- | --- |

where: $f$ is the frequency and$f_{s}$is the sampling rate.

The non-normalized DTF is defined as follows [5 43]:

| $\mathbf{H}(f)=\left\vert\frac{1}{1-\sum_{j=1}^{p} \mathbf{A}_{j}\exp\left(\frac{-2\pi\sqrt{-1}jf}{f_{s}}\right)}\right\vert$ | (10) |
| --- | --- |

The element $h_{il}(f)$of matrix $\mathbf{H}(f)$ is the measure of directional information flow from channel *l* to channel *i* [5].

One of the methods of estimating the MVAR model is the Yule-Walker Algorithm [6]. It allows for computation of MVAR coefficients based on the covariance matrix of the signal $\vec{x}(i)$, as follows. Multiplying the equation (8) from the right by $\vec{x}^{\text{T}}(i-j)$ for $j=1,\ldots,p$, and then taking the exceptional value, one can obtain:

| $\mathbf{R}(-j)=\sum_{j=1}^{p} \mathbf{A}_{j}\mathbf{R}(-j+1)$ | (11) |
| --- | --- |

where: T denotes transposition, is the covariance matrix of process $\vec{x}(i)$at lag *j*. In this work we use the following estimator of the covariance matrix $\mathbf{R}(-j)$:

|  | (12) |
| --- | --- |

Multiple realizations were available In our experiment. Thus we used a modification of the Yule-Walker Algorithm [7], where the final estimator of the covariance matrix (see equation (12)) was obtained by averaging EEG covariance matrixes calculated for each repetition. DTF were calculated for 3 subsequent time windows: 0-200 ms, 200-400 ms, and 400-600 ms.

***References***

1. Makeig S. Auditory event-related dynamics of the EEG spectrum and effects of exposure to tones. Electroencephalogr. Clin Neurophysiol. 1993;86:283–293.

2. Kronland-Martinet R, Morlet J, Grossmann A. Analysis of sound patterns through wavelet transforms. Int J Pattern Recogn. 1987;1:273–302.

3. Challis RE, Kitney RI. Biomedical signal processing. Part 3. The power spectrum and coherence function. Med Biol Eng Comput. 1991;29:225–241.

4. Andrew C, Pfurtscheller G. Event-related coherence as a tool for studying dynamic interaction of brain regions. Electroencephalogr Clin Neurophysiol. 1996;98:144–148.

5. Kamiński MJ, Blinowska KJ. A new method of the description of the information flow in the brain structures. Biol Cybern. 1991;65:203–210.

6. Marple SL. Digital Spectral Analysis With Applications (Prentice-Hall Series in Signal Processing; Englewood Cliffs, NJ: Prentice Hall; 1987.

7. Ding M, Bressler SL, Yang W, Liang H. Short-window spectral analysis of cortical event-related potentials by adaptive multivariate autoregressive modeling: data preprocessing, model validation, and variability assessment. Biol Cybern. 2000;83:35–45.
