# Supplementary material for: Name recognition in autism: EEG evidence of altered patterns of brain activity and connectivity
Source: Mol Autism. 2016 Sep 6;7(1):38. doi: 10.1186/s13229-016-0102-z (PMC5012044; doi:10.1186/s13229-016-0102-z)
Supplement: Additional file 2: — Figures illustrating ERD/S, coherence, and DTF averaged across groups and experimental conditions. (DOCX 1165 kb) [file 13229_2016_102_MOESM2_ESM.docx]

**Figures illustrating results of ERD/S, coherence and DTF calculations for averaged groups and experimental conditions**

**Figure A1.** ERD/S **
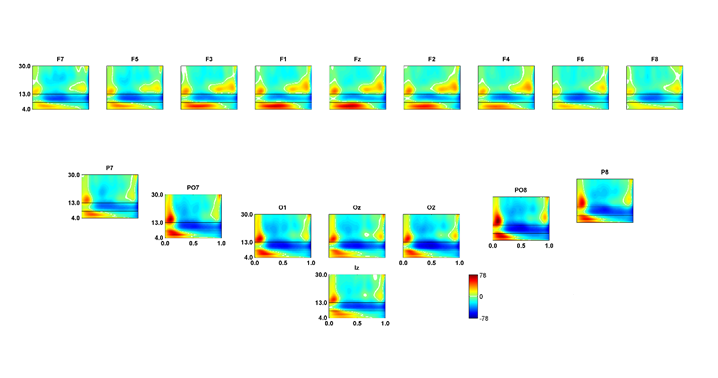
**

**Figure A2.** Coherence
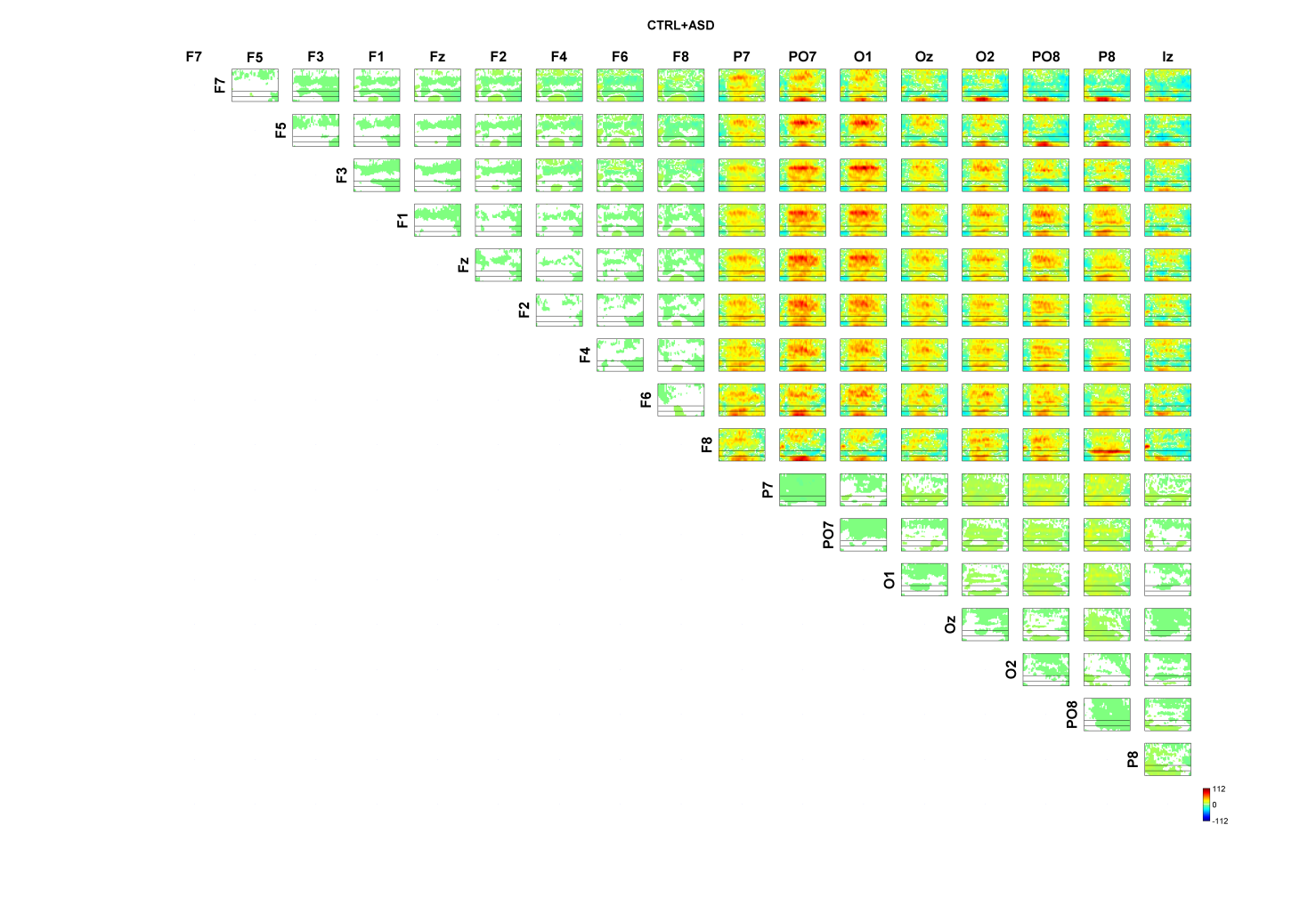


**Figure A3.** DTF in 0-200 ms time window
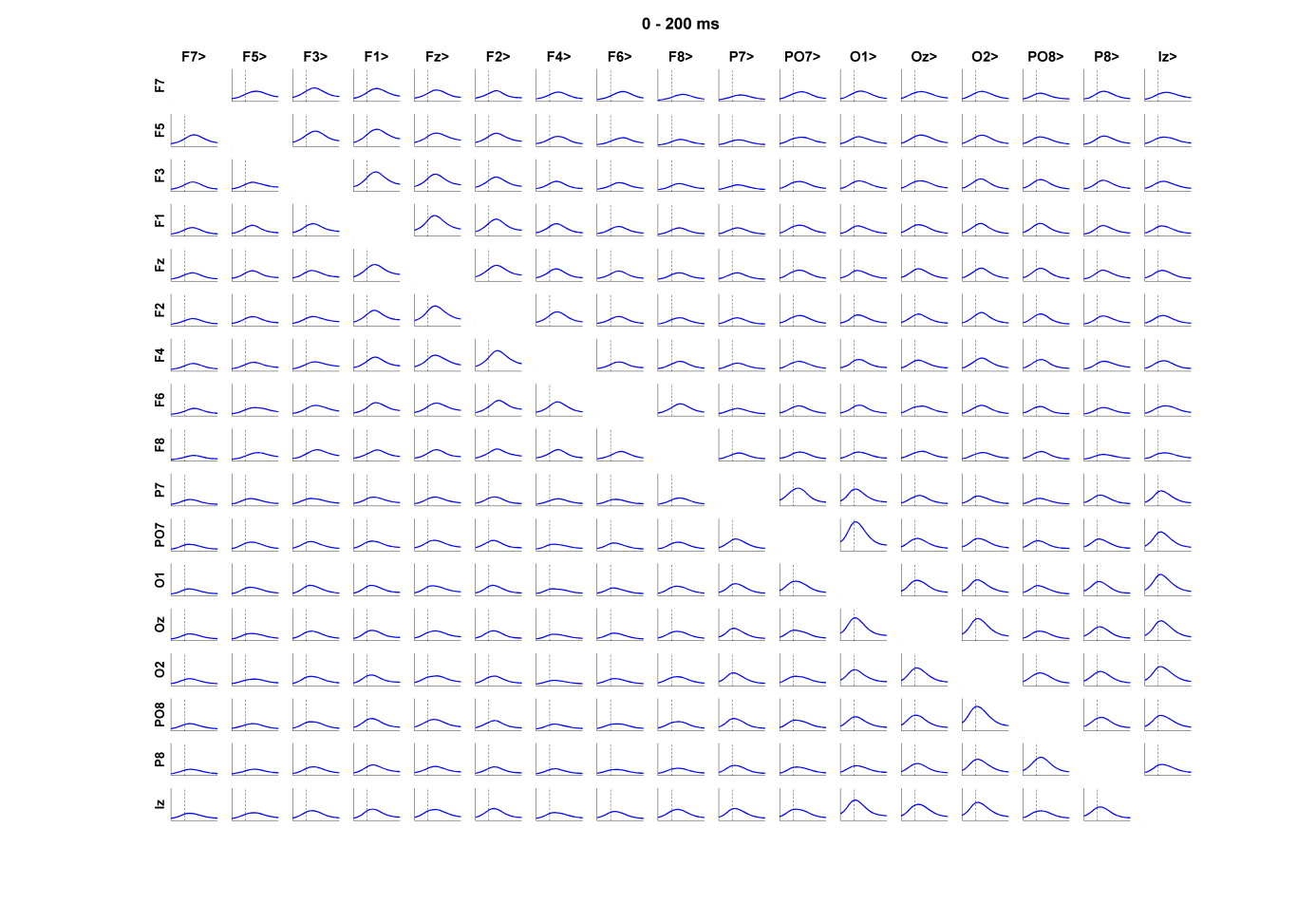


**Figure A4.** DTF in 200-400 ms
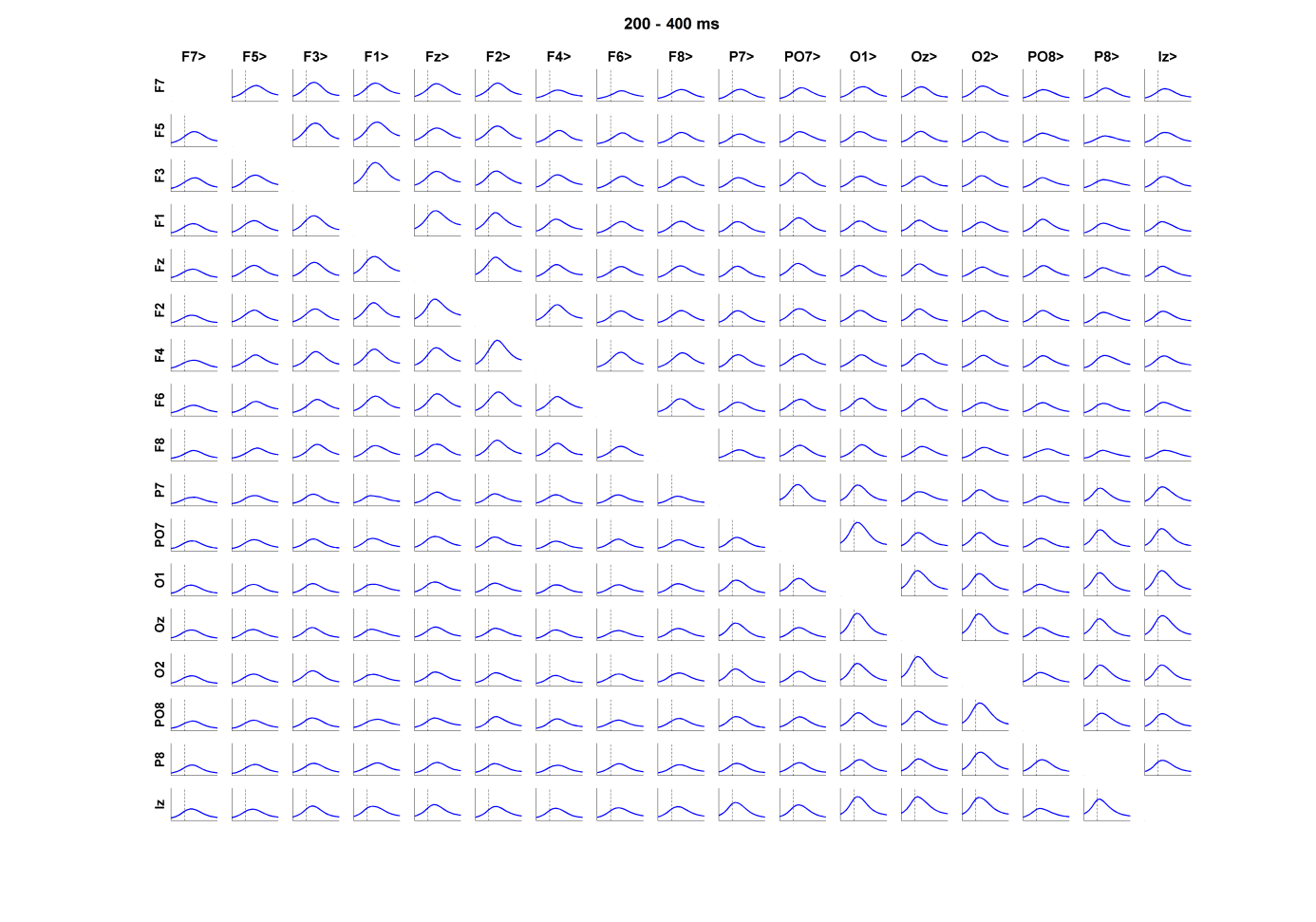


**Figure A5.** DTF in 400-600 ms time window **
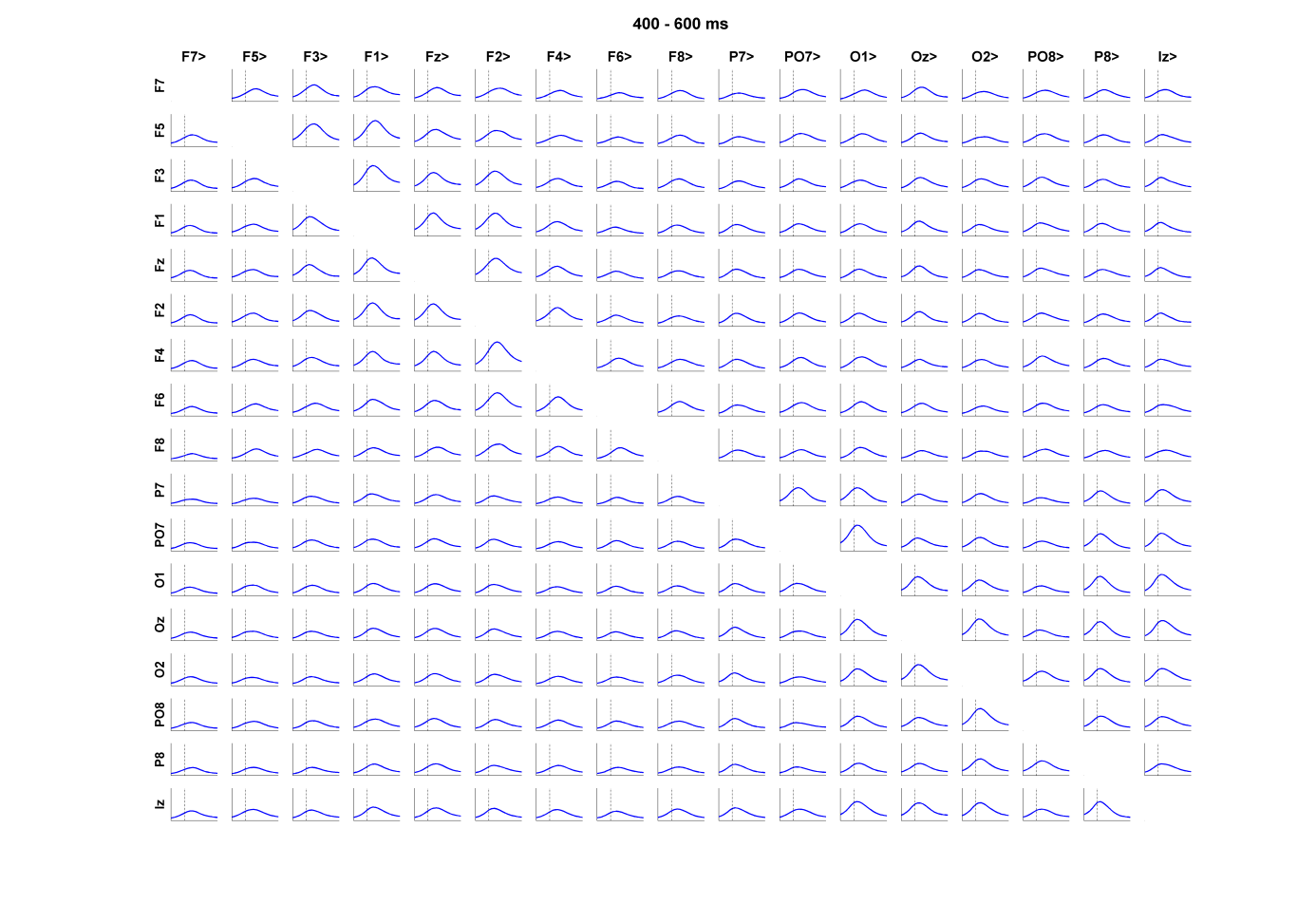
**
