# Supplementary material for: Name recognition in autism: EEG evidence of altered patterns of brain activity and connectivity
Source: Mol Autism. 2016 Sep 6;7(1):38. doi: 10.1186/s13229-016-0102-z (PMC5012044; doi:10.1186/s13229-016-0102-z)
Supplement: Additional file 3: — Figures illustrating complete results of ERD/S, coherence, and DTF calculations. (DOCX 3256 kb) [file 13229_2016_102_MOESM3_ESM.docx]

**Figures illustrating complete results of ERD/S, coherence, and DTF calculations**

**Figure A6.** ERD/S averaged across all categories of names in the control group (upper panel) and in the group of individuals with ASD (lower panel) for 17 frontal and parietal-occipital electrode sites.

**
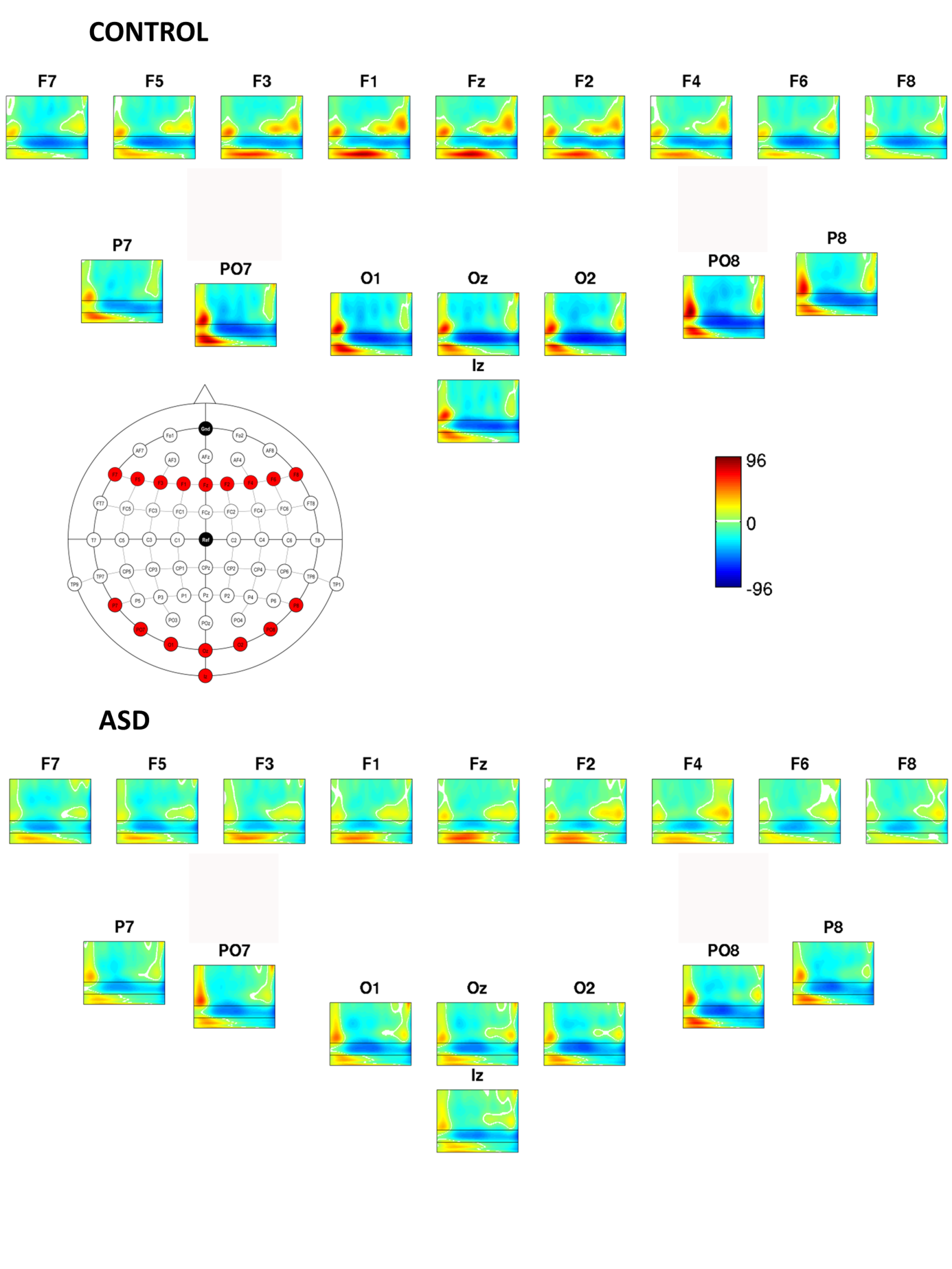
**

**Figure A7**. Time course of coherence averaged across all categories of names in the control group (upper panel) and in the ASD group (lower panel) for 17 frontal and parietal-occipital electrode sites.


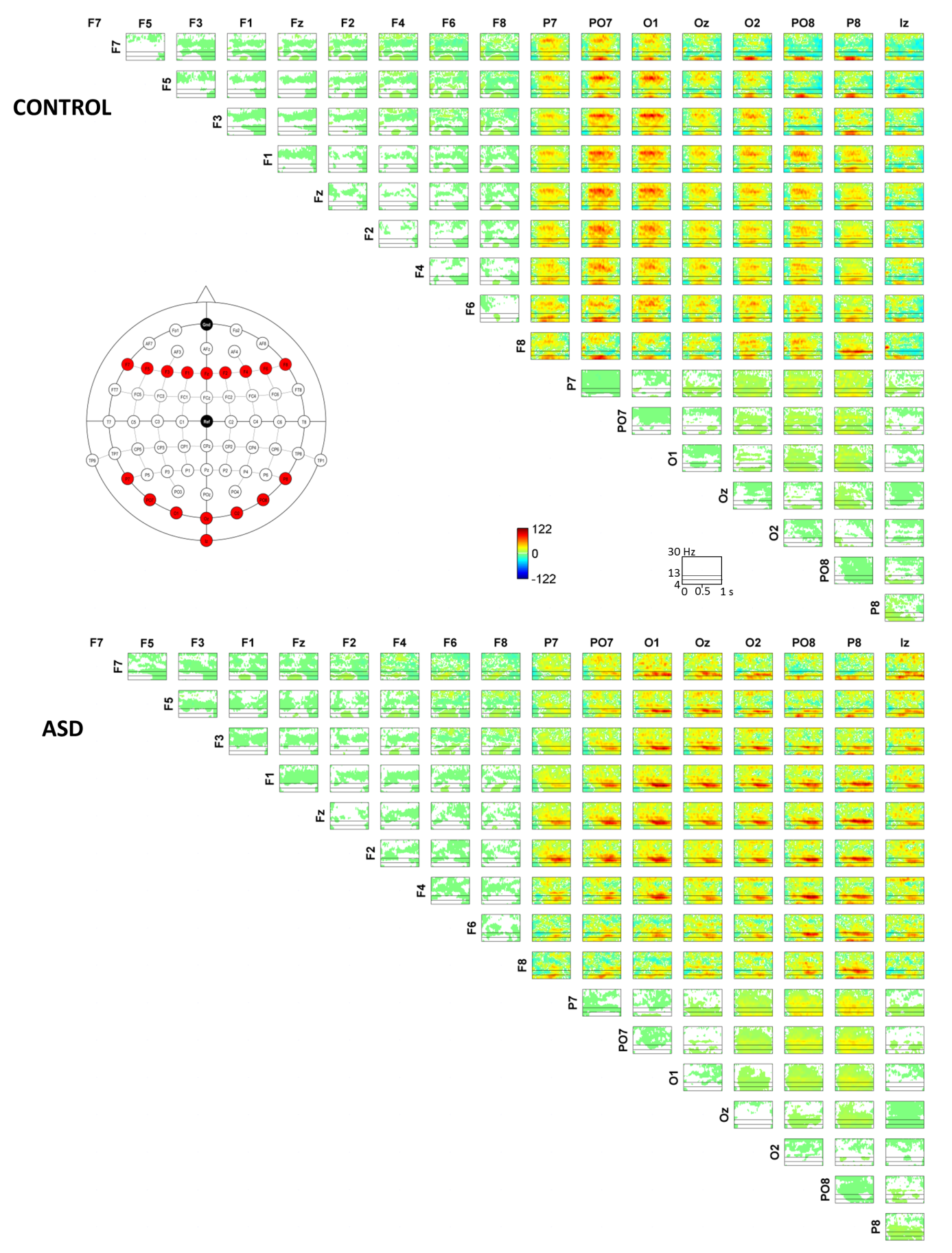


**
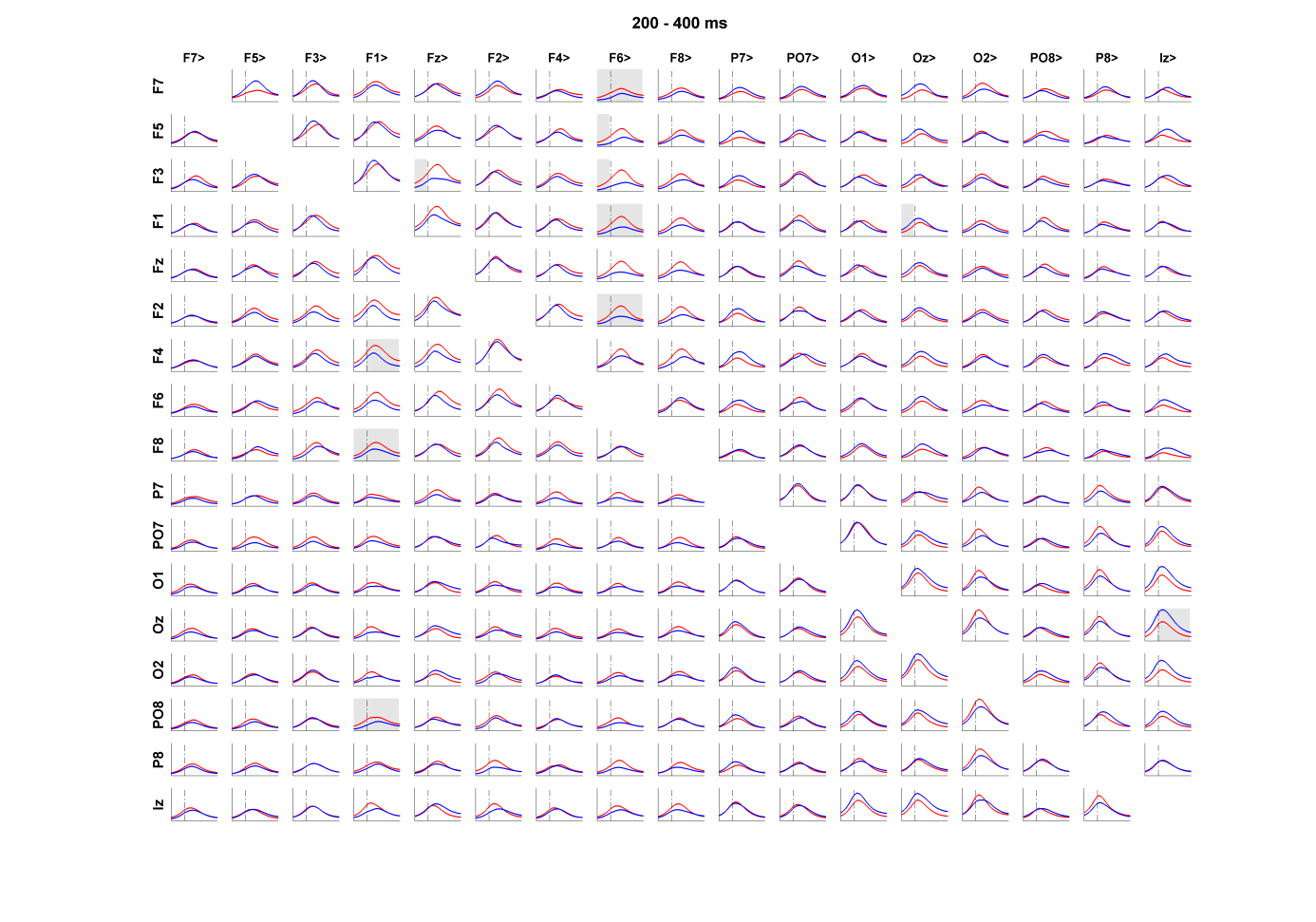

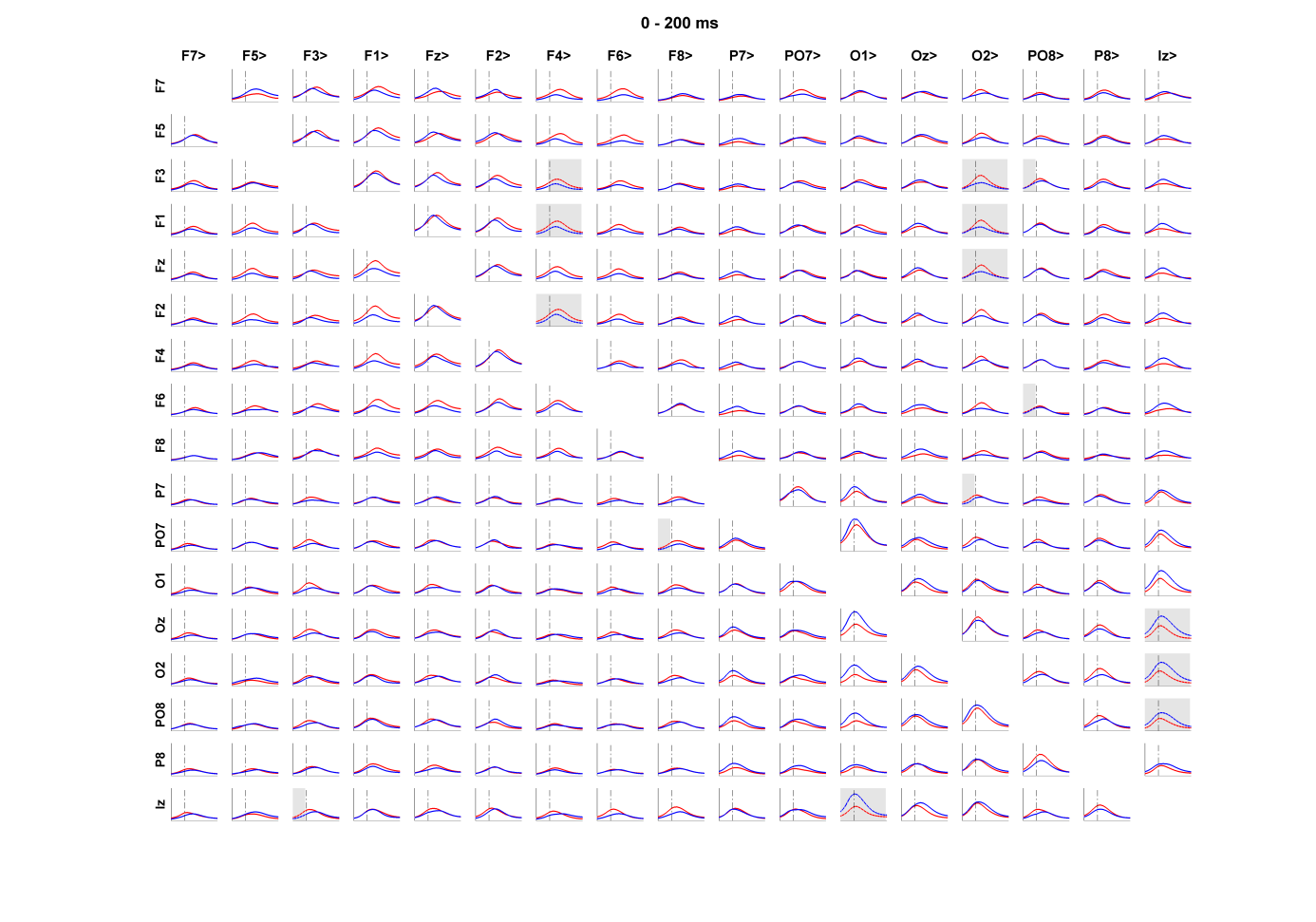
Figure A8.** DTFs in the control group (red line) and in the ASD group (blue line) for the 3 consecutive time windows: 0-200 ms, 200-400 ms, and 400-600 ms. Calculations were done for frontal and parietal-occipital electrode sites. Gray color rectangles indicate significant differences between ASD and control groups (uncorrected p values). **
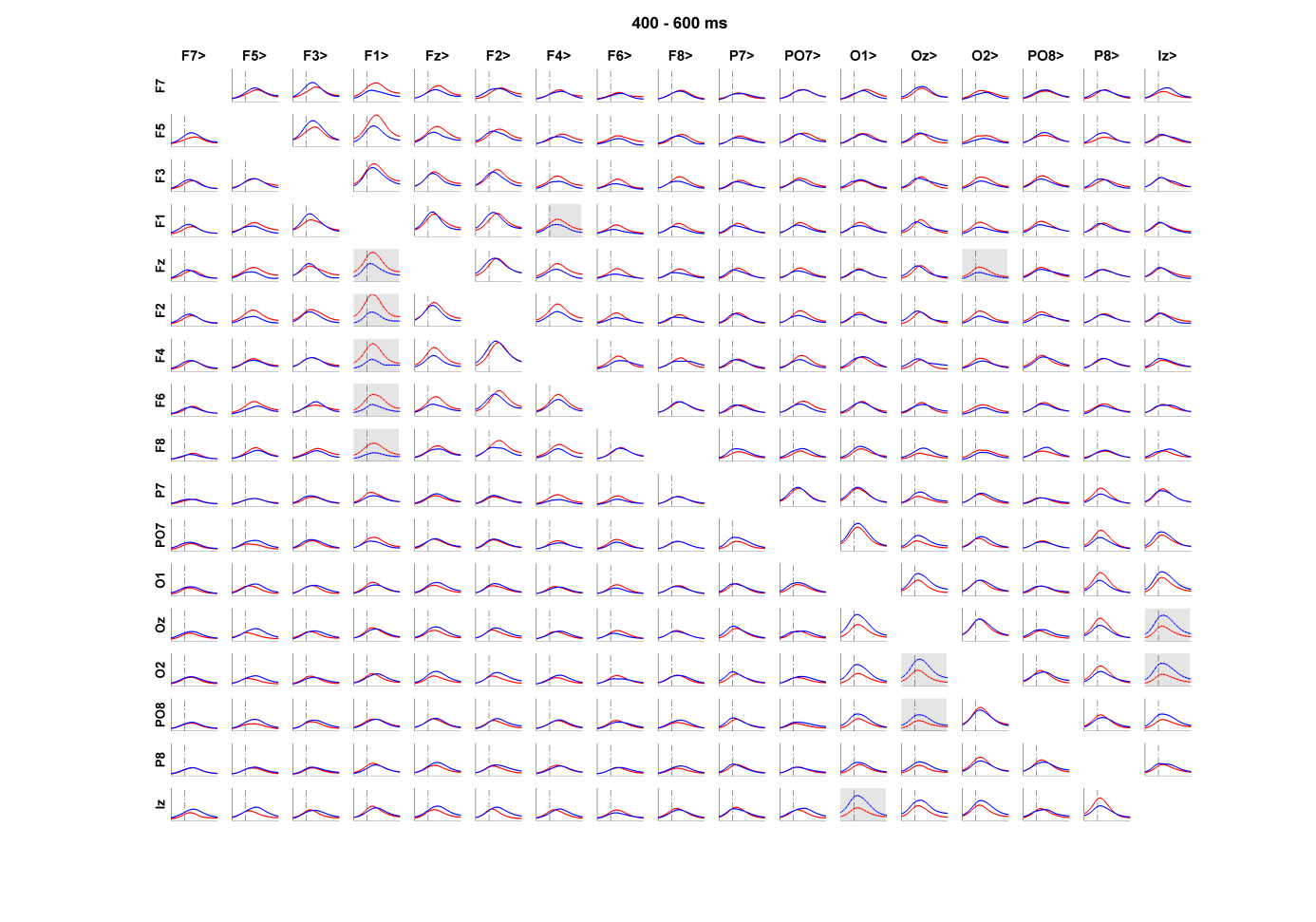
**
