# Supplementary material for: Name recognition in autism: EEG evidence of altered patterns of brain activity and connectivity
Source: Mol Autism. 2016 Sep 6;7(1):38. doi: 10.1186/s13229-016-0102-z (PMC5012044; doi:10.1186/s13229-016-0102-z)
Supplement: Additional file 4: — Tables with ERD/S, coherence, and DTF results that did not retain the significance after FDR corrections. (DOCX 45 kb) [file 13229_2016_102_MOESM4_ESM.docx]

**Tables with ERD/S, coherence, and DTF results**

**Table A1.** Results of ERD/S analyses. CTRL > ASD indicates stronger effects in the control group than in the group of individuals with ASD. ASD > CTRL indicates stronger effects in the group of individuals with ASD than in the control group. F – frontal electrodes; PO – parietal-occipital electrodes; O, I – occipital electrodes. Odd numbers (e.g. F1) - electrodes located over the left side of the head; even numbers (e.g. F8) - electrodes located over the right side of the head; ‘z’ (e.g. Iz) - electrodes located at the midline. None of effects retained significance after applying FDR corrections.

| **frequency** |  | **electrode** | **F values** | ***p* values**  **(uncorrected)** | **η_p_^2^** |
| --- | --- | --- | --- | --- | --- |
| **theta** | CTRL > ASD | O1  PO7 | 4.416  4.854 | 0.045  0.036 | 0.136  0.148 |
|  | ASD > CTRL | – | – | – | – |
| **alpha** | CTRL > ASD | – | – | – | – |
|  | ASD > CTRL | O2 | 3.967 | 0.050 | 0.124 |
| **beta** | CTRL > ASD | F6  F7  F8  Iz | 5.053  5.752  6.796  7.337 | 0.033  0.023  0.014  0.011 | 0.153  0.170  0.195  0.208 |
|  | ASD > CTRL | – | – | – | – |

**Table A2.** Task-related connectivity revealed by the coherence analysis. CTRL > ASD indicates stronger connections in the control group than in the group of individuals with ASD. ASD > CTRL indicates stronger connections in the group of individuals with ASD than in the control group. F – frontal electrodes; P – parietal electrodes; PO – parietal-occipital electrodes; O – occipital electrodes. Odd numbers (e.g. F1) - electrodes located over the left side of the head; even numbers (e.g. F8) - electrodes located over the right side of the head; ‘z’ (e.g. Fz) - electrodes located at the midline. ‘*’ marks effects significant after applying FDR correction.

| **frequency** |  | **pair of electrodes** | **F values** | ***p* values**  **(uncorrected)** | **η_p_^2^** |
| --- | --- | --- | --- | --- | --- |
| **theta** | CTRL > ASD | F1 – P8  Fz – P8  F3 – P8 | 4.898  3.647  3.479 | 0.035  0.066  0.073 | 0.149  0.115  0.073 |
|  | ASD > CTRL | – | – | – |  |
| **alpha** | CTRL > ASD | – | – | – |  |
|  | ASD > CTRL | F3 – PO8  F3 – P8 | 5.540  3.752 | 0.026  0.060 | 0.165  0.118 |
| **beta** | CTRL > ASD | F4 – PO7  F6 – PO7  F4 – PO8  F4 – O1  F6 – O1  F8 – O1  F4 – O2  Fz – O2  F1 – O2  F2 – O2  F6 – O2  F8 – O2 | 6.424  5.955  3.758  5.447  10.319  4.883  4.361  11.728  8.262  12.979  5.307  5.720 | 0.017  0.021  0.060  0.025  0.003*  0.035  0.046  0.002*  0.008*  0.001*  0.029  0.024 | 0.187  0.175  0.118  0.163  0.269  0.148  0.135  0.295  0.228  0.317  0.159  0.164 |
|  | ASD > CTRL | – | – | – |  |

**Table A3.** Task-related connectivity patterns revealed by Directed Transfer Function within the beta band. CTRL > ASD indicates stronger connections in the control group than in the ASD group. ASD > CTRL indicates stronger connections in the ASD group than in the control group. F – frontal electrodes; P – parietal electrodes; PO – parietal-occipital electrodes; O – occipital electrodes. Odd numbers (e.g. F1) - electrodes located over the left side of the head, even numbers (e.g. F8) - electrodes located over the right side of the head, and the ‘z’ letter (e.g. Fz) - electrodes located in the midline. Arrow indicates directionalities of connections (e.g. O2→F1, from O2 to F1); ‘*’ marks results significant after applying FDR correction.

| **time window** |  | **13-18 Hz** | ***p* values**  **(uncorrected)** | **18-30 Hz** | ***p* values**  **(uncorrected)** |
| --- | --- | --- | --- | --- | --- |
| **0-200 ms** | CTRL > ASD | O2→F1  O2→F3  O2→Fz  PO8→F3  PO8→F6  F8→PO7  F3→Iz  O2→P7  F4→F1  F4→F2 | 0.03  0.02  0.04  0.02  0.02  0.02  0.01  0.02  0.03  0.03 | O2→F1  O2→F3  O2→Fz  F4→F1  F4→F3  F4→F2 | 0.01  < 0.001*  0.01  0.01  0.01  0.01 |
|  | ASD > CTRL | Iz→Oz  Iz→O2  Iz→PO8  O1→Iz | 0.02  0.02  0.04  0.05 | Iz→Oz  Iz→O2  Iz→PO8  O1→Iz | 0.01  0.02  0.05  0.01 |
| **200-400 ms** | CTRL > ASD | F6→F1  F6→F2  F6→F3  F6→F5  F6→F7  Fz→F3  F1→F8  F1→PO8 | 0.02  0.03  0.01  < 0.001*  0.01  0.01  0.03  0.001 | F6→F1  F6→F2  F6→F7  F1→F4  F1→F8 | 0.04  0.03  0.04  0.01  0.04 |
|  | ASD > CTRL | Iz→Oz  Oz→F1 | 0.05  0.02 | Iz→Oz | 0.01 |
| **400-600 ms** | CTRL > ASD | F1→Fz  F1→F2  F1→F4  F1→F6  F1→F8  O2→Fz | 0.03  < 0.001*  < 0.001*  < 0.001*  < 0.001*  0.03 | F1→Fz  F1→F2  F1→F4  F1→F6  F1→F8  F4→F1  O2→Fz | < 0.001*  < 0.001*  < 0.001*  < 0.001*  < 0.001*  0.02  0.02 |
|  | ASD > CTRL | Iz→Oz  Iz→O2  Oz→O2  Oz→PO8  O1→ Iz | 0.03  0.01  0.01  0.02  0.04 | Iz→Oz  Iz→O2  Oz→O2  Oz→PO8  O1→ Iz | 0.02  0.02  < 0.001*  0.05  0.05 |
